# Effect of a twin-emitter design strategy on a previously reported thermally activated delayed fluorescence organic light-emitting diode

**DOI:** 10.3762/bjoc.17.197

**Published:** 2021-12-08

**Authors:** Ettore Crovini, Zhen Zhang, Yu Kusakabe, Yongxia Ren, Yoshimasa Wada, Bilal A Naqvi, Prakhar Sahay, Tomas Matulaitis, Stefan Diesing, Ifor D W Samuel, Wolfgang Brütting, Katsuaki Suzuki, Hironori Kaji, Stefan Bräse, Eli Zysman-Colman

**Affiliations:** 1 Organic Semiconductor Centre, EaStCHEM School of Chemistry, University of St Andrews, St Andrews, Fife, KY16 9ST, UK; 2 Institute of Organic Chemistry, Karlsruhe Institute of Technology (KIT), Fritz-Haber-Weg 6, 76131 Karlsruhe, Germany; 3 Institute for Chemical Research, Kyoto University, Uji, Kyoto 611-0011, Japan; 4 Experimental Physics IV, Institute of Physics, University of Augsburg, Universitätstrasse. 1, 86159 Augsburg, Germany; 5 Organic Semiconductor Centre, SUPA, School of Physics and Astronomy, University of St Andrews, North Haugh, St Andrews, KY16 9SS, UK; 6 Institute of Biological and Chemical Systems – Functional Molecular Systems (IBCS-FMS), Karlsruhe Institute of Technology (KIT), Hermann-von-Helmholtz-Platz 1, D-76344 Eggenstein-Leopoldshafen, Germany

**Keywords:** blue emitters, dimer, indolocarbazole, orientation, outcoupling effect, solution-processed OLEDs, TADF emitters, triazine

## Abstract

In this work we showcase the emitter **DICzTRZ** in which we employed a twin-emitter design of our previously reported material, **ICzTRZ**. This new system presented a red-shifted emission at 488 nm compared to that of **ICzTRZ** at 475 nm and showed a comparable photoluminescence quantum yield of 57.1% in a 20 wt % CzSi film versus 63.3% for **ICzTRZ**. The emitter was then incorporated within a solution-processed organic light-emitting diode that showed a maximum external quantum efficiency of 8.4%, with Commission Internationale de l’Éclairage coordinate of (0.22, 0.47), at 1 mA cm^−2^.

## Introduction

Organic thermally activated delayed fluorescence (TADF) materials have elicited tremendous excitement as an alternative to phosphorescent complexes in organic light-emitting diodes (OLEDs) because these organic compounds can also achieve a theoretical 100% internal quantum efficiency (IQE) but do not require the use of scarce, noble metals [1–2]. Since the luminescence in an OLED is achieved through the radiative decay of electrically generated excitons, high-efficiency devices must be able to harvest both the 25% singlet and 75% triplet excitons to produce light [3]. Distinct from phosphorescent compounds, TADF molecules harvest triplet excitons by converting them into emissive singlets via a reverse intersystem crossing (RISC) mechanism. This mechanism is operational when the energy gap (Δ*E*_ST_) between the lowest-lying singlet and triplet excited states (S_1_ and T_1_) is sufficiently small and spin-orbit coupling (SOC) is non-negligible [4–7]. This small Δ*E*_ST_ can be achieved by spatially separating the highest occupied molecular orbital (HOMO) and the lowest unoccupied molecular orbital (LUMO), thereby reducing the exchange integral of these two orbitals determining the energies of the S_1_ and T_1_ states relative to the ground state. The spatial separation of the HOMO and LUMO on donor and acceptor, respectively, will result in an S_1_→S_0_ transition with predominantly charge transfer (CT) character. Highly twisted donor–acceptor architectures are typically employed to realize small Δ*E*_ST_ [4,8]. SOC can be enhanced by ensuring that the nature of the S_1_ and T_1_ states is different, for example by additionally involving a third (local) triplet state with different symmetry, because otherwise SOC vanishes when the orbital types for these two states are the same, according to El-Sayed’s rule [9].

Designing a molecule able to achieve RISC and the desired 100% IQE is just the first step toward an efficient OLED since the light needs to escape the device. A device is composed of a stack of several layers of organic semiconductor materials, each possessing different refractive indices, sandwiched between two electrodes. Depending on the angle of emission of the light with respect to the plane of the device, total internal reflection at the organic-glass as well as the glass-air interfaces can occur as can coupling to surface plasmon polaritons (SPP) at the interface with the cathode, all contributing to decreasing the external quantum efficiency (EQE) of the device. A compound will emit light perpendicular to its transition dipole moment (TDM), quantified by the anisotropy factor, *a*. Controlling the orientation of the TDM to lie horizontally in the film (where 1 − *a* is the fraction of horizontally aligned TDMs) will lead to a maximized amount of light exiting the device. However, when the transition dipoles of the emitter are randomly oriented then only around 20% of the light can escape the device [10].

**Figure 1:**
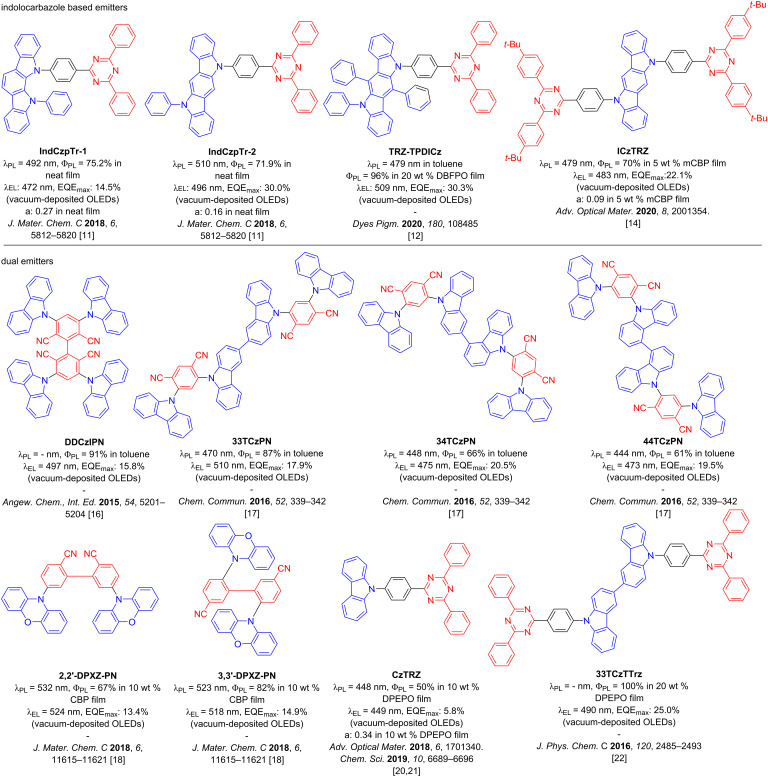
Molecular structures of emitters.

Indolocarbazole (**ICz**)-based emitters have been recently employed in several high-performance and highly horizontally oriented materials. ICz acts as a weak, planar, and rigid donor [11–14]. Examples of compounds incorporating an ICz unit include reports from Xiang et al. with the emitters **IndCzpTr-1** and **IndCzpTr-2** [11], and Maeng et al. with the emitter **TRZ-TPDICz** [12] (see Figure 1). In the doped film, **IndCzpTr-1** and **IndCzpTr-2** present high photoluminescence quantum yields, Φ_PL_, of 75.2% and 71.9%, respectively, and delayed fluorescence lifetimes, τ_d_, of 25.48 μs and 34.31 μs, respectively. The devices produced with these materials reached maximum external quantum efficiencies (EQE_max_) values of 14.5% and 30% at low brightness, but efficiency roll-off was significant, with EQE at 100 cd m^−2^, EQE_100_, of 11.0% and 15.3% for the OLEDs with **IndCzpTr-1** and **IndCzpTr-2**, respectively. The addition of two phenyl units on the ICz in **TRZ-TPDICz** increased the donor strength and led to Φ_PL_ of near unity (96%) and a much shorter τ_d_ of 8.57 μs in 20 wt % DBFPO film (DBFPO = 2,8-bis(diphenylphosphine oxide)dibenzofuran). The device made from this material has a very high EQE_max_ of 30.3%, which decreases to 18.4% at 1000 cd m^−2^; the use of a stronger donor in **TRZ-TPDICz** results in a red-shift of the electroluminescence, compared to **IndCzpTr-1** and **IndCzpTr-2** (the electroluminescence maximum wavelength, λ_EL_ of 472 nm and 496 nm for **IndCzpTr-1** and **IndCzpTr-2**, respectively, against λ_EL_ of 509 nm for **TRZ-TPDICz**). In our previous work, we presented the first example of a di-functionalized ICz-based emitter **ICzTRZ** [13–14], that presented nearly complete horizontal orientation in a wide number of host matrices. The best combination of properties was obtained in mCBP as a host, with the photoluminescence maximum wavelength, λ_PL_ of 479 nm, Φ_PL_ of 70%, and a τ_d_ of 121.1 μs for the vacuum-deposited doped film. The anisotropy factor (*a*) in 5 wt % mCBP film is 0.09, indicating a very high degree of horizontal orientation (91%), which together with the high Φ_PL_ led to a high-performing device with EQE_max_ of 22.1% (Figure 1).

It has been documented in the literature that some multichromophore emitters show enhanced molar extinction coefficients of absorption and high Φ_PL_ [15–18]. This led to OLEDs employing dual or multi emitter-designed compounds to show much improved EQE_max_ compared to devices with their single-emitter counterparts (Figure 1), albeit with a red-shifted emission [16–19]. The advantages of the dual-emitter design are best illustrated by the cross-comparison of **CzTRZ** [20–21], a molecule that did not present any TADF and thus the OLED showed a low EQE_max_ of 5.8%, while the emitter, **33TCzTTrz** [22], is TADF and the OLED showed a much superior EQE_max_ of 25.0%. There is a significant red-shift of the electroluminescence, with λ_EL_ going from 449 nm for **CzTRZ** to 490 nm for **33TCzTTrz**.

**Figure 2:**
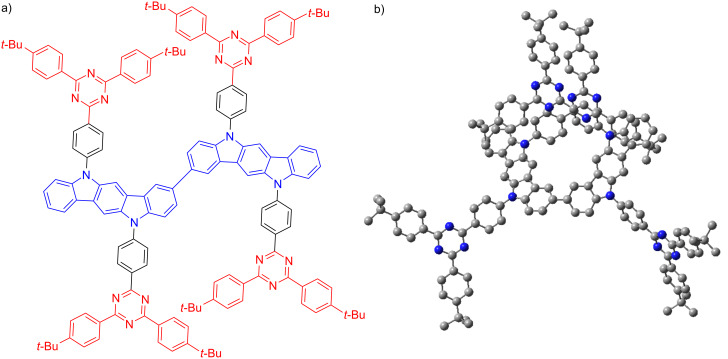
a) Molecular structure and b) optimized DFT-calculated geometry of **DICzTRZ**. Hydrogen atoms are omitted for clarity.

In this work, we utilized a similar strategy to assess the change in optoelectronic properties and device performance of the compound **DICzTRZ** (Figure 2) compared to our recently reported **ICzTRZ** study [14]. We note that the effective doubling of the molecular weight necessitates that we fabricate solution-processed devices. Importantly, solution-processed films tend to present isotropic orientation [10] due to the slower deposition times coupled with higher degree of freedom of movement in the solution, unlike the orientation of the emitter in vacuum-deposited films, which occurs only at the surface of the film where the emitter orientation is then “frozen” into place once additional layers of material have covered it. While this loss of controlled orientation in the solution-processed film is true for small molecules, polymers and other high molecular weight emitters can show at least some degree of orientation in solution-processed films. For instance, Senes et al. [23–24]. showed that the **OPVn** series of polymers exhibited higher horizontal orientation by increasing the length of the polymer chain, and by extension the molecule. Considering the high degree of horizontal orientation that **ICzTRZ** already showed in vacuum-deposited films (anisotropy factor of 0.09 in 10 wt % film of mCBP) and the high molecular weight of **DICzTRZ**, we hypothesized that **DICzTRZ** may also present horizontal orientation in the film and subsequently improve light outcoupling in the device.

## Results and Discussion

### Synthesis

The oxidative coupling conditions for the synthesis of carbazole dimers were initially applied to access the dimer of **ICzTRZ** [25–26]. Treating **ICzTRZ** with FeCl_3_ in dichloromethane (DCM) at room temperature for 12 hours did not lead to any product formation. However, when the temperature was increased to 40 °C, **DICzTRZ** was formed and was isolated in a yield of 20%, while increasing the temperature to 60 °C resulted in complete consumption of the starting material and **DICzTRZ** was isolated in 66% yield. The identity and purity of **DICzTRZ** were determined by a combination of NMR spectroscopy, mass spectrometry, and IR spectroscopy.

### Theoretical calculations

**Figure 3:**
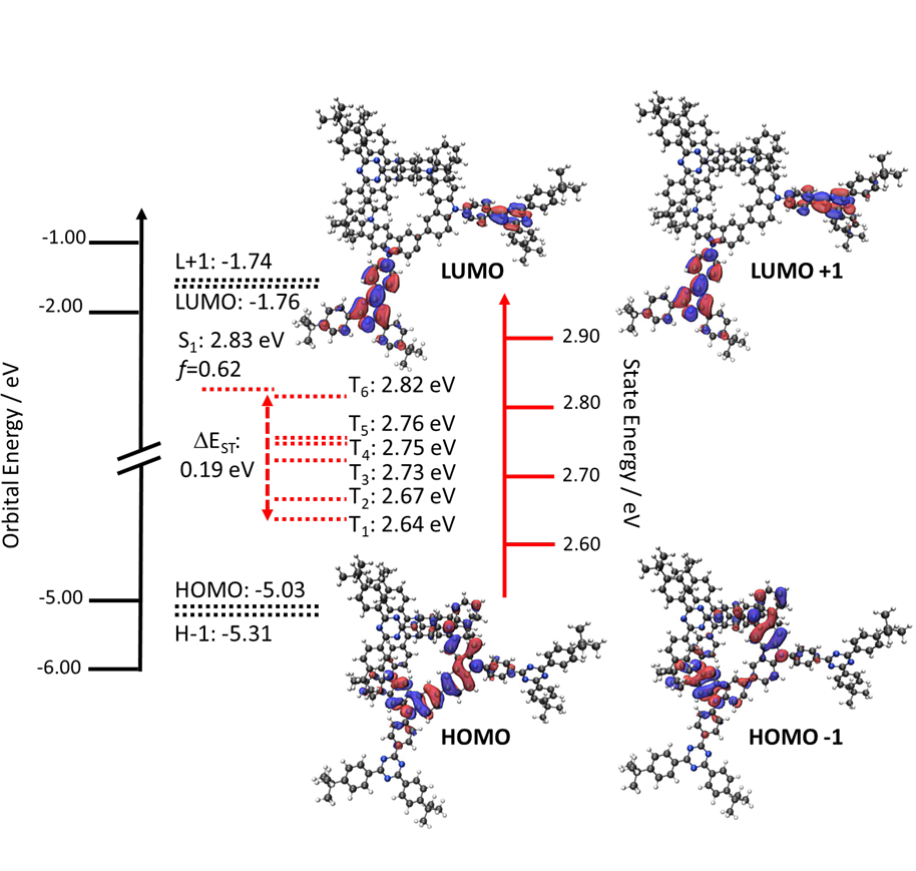
HOMO, HOMO–1 (H–1), LUMO, and LUMO+1 (L+1) electron density distributions (isovalue: 0.02) and energy levels, excited state energy levels.

Density functional theory (DFT) and time-dependent DFT (TD-DFT) calculations in the gas phase at the PBE0/6-31G(d,p) level reveal the potential of **DICzTRZ** as a TADF material. The nature of the S_1_ and T_1_ states and their corresponding energies were then obtained using the Tamm–Dancoff approximation [27] to TD-DFT (TDA-DFT). **DICzTRZ** possesses a Δ*E*_ST_ of 0.19 eV, comparable to 0.22 eV obtained for **ICzTRZ** at the same level of theory. We can observe a slightly stabilized S_1_ energy of 2.83 eV (2.92 eV for **ICzTRZ**) and T_1_ energy of 2.64 eV (2.70 eV for **ICzTRZ**) [14] compared to those of **ICzTRZ**. Compared to **ICzTRZ,** there is a much higher density of intermediate triplet states between S_1_ and T_1_, which is expected to enhance the efficiency of the RISC process due to the presence of increased spin-vibronic coupling [28–33]. The permanent dipole moment (PDM) of **DICzTRZ** is substantial increased to 2.1 Debye compared to that in **ICzTRZ** (0.3 Debye); however, both the transition dipole moment (TDM) and oscillator strength (*f*) are slightly smaller (TDM = 7.6 Debye and *f* = 0.62) than the values calculated for **ICzTRZ** (TDM = 7.9 Debye and *f* = 0.72). **DICzTRZ** shows a shallower HOMO at −5.03 eV, reflective of a certain degree of conjugation between the two indolocarbazole moieties, compared to the HOMO of **ICzTRZ** (−5.19 eV). The LUMO level remains essentially unchanged (−1.76 eV for **DICzTRZ** vs −1.75 eV for **ICzTRZ**) since the electronic environment surrounding the *t-*Bu-triazine remains essentially unperturbed (Figure 3).

### Optoelectronic properties

**Figure 4:**
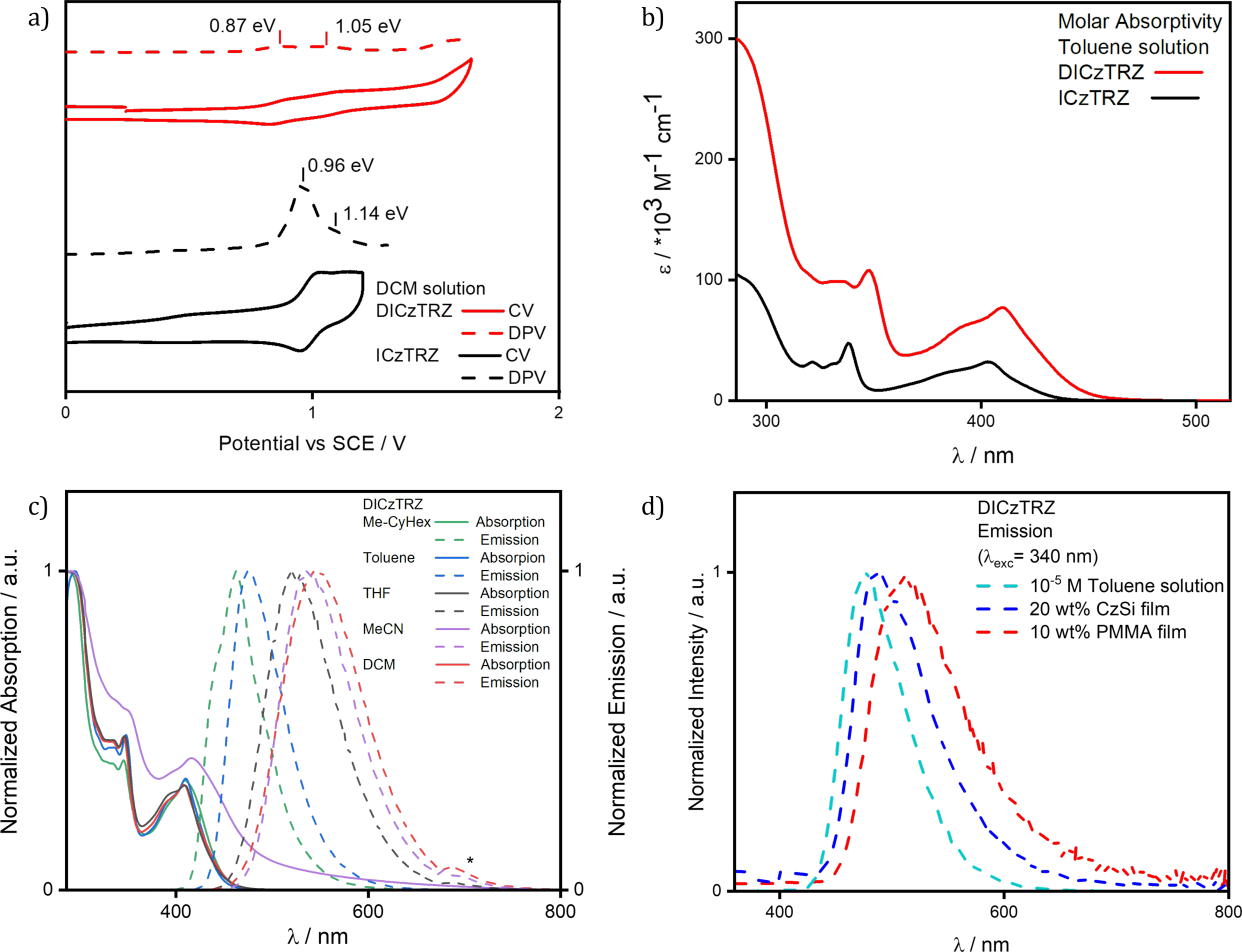
a) Cyclic voltammetry (CV) and differential pulse voltammetry (DPV) of **DICzTRZ** in DCM (scan rate = 100 mV/s). b) UV–vis absorption spectrum of **DICzTRZ** in 10^−5^ M toluene solution. c) Ground and excited state solvatochromism study of **DICzTRZ** (excitation wavelength, λ_exc_, = 340 nm, * = second harmonic of the excitation source); d) emission spectra of **DICzTRZ** in 10^−5^ M toluene solution (cyan), 20 wt % CzSi film (blue), and 10 wt % PMMA film (red), (λ_exc_ = 340 nm).

The electrochemical properties of the two materials were studied in degassed DCM with tetra-*n*-butylammonium hexafluorophosphate as the electrolyte and Fc/Fc^+^ as the internal reference, data are reported versus a saturated calomel electrode (SCE). In both **DICzTRZ** and **ICzTRZ** [14] we observed a reversible oxidation wave with respective oxidation potential (*E*_ox_) at 0.87 V and 0.96 V vs SCE (Figure 4a). Both compounds also present a second oxidation wave that is more prominent and cathodically shifted for **DICzTRZ** at 1.05 V, compared to 1.14 V for **ICzTRZ**. No reduction wave is observed for **DICzTRZ**. The HOMO value calculated from the oxidation potential obtained from differential pulse voltammetry (DPV), is −5.21 eV, which is stabilized compared to that predicted from DFT (*E*_HOMO_: −5.03 eV); however, the less positive oxidation potentials in **DICzTRZ** versus **ICzTRZ** does align with the predictions obtained by DFT.

The UV–vis absorption spectrum of **DICzTRZ**, while slightly red-shifted and with higher molar absorptivity (as was the case for previously published multichromophore materials) [15–17,34–35], coincides closely with the one from **ICzTRZ** [14] (Figure 4b) and also with other indolocarbazole-based compounds [11]. The nearly identical profile leads us to conclude that the character of the transitions is likely to be very similar to those associated with **ICzTRZ**. The two absorption bands located between 330 and 350 nm are ascribed to the LE transitions within the diindolocarbazole donor. The two lower energy and lower absorptivity bands at 390 nm and 410 nm are both assigned to CT-type transitions (Figure 4b).

Solvatochromic studies for **DICzTRZ** show that the PDM of the ground state structure is small and so the absorption spectrum is essentially not affected by changes in polarity, while the excited state shows the characteristic positive solvatochromism associated with an emission from a CT state (λ_PL_ going from 462 nm in the least polar methylcyclohexane to 548 nm in the most polar dichloromethane). From the previously calculated HOMO level determined from DPV and the optical gap obtained from the intersection of the normalized absorption and emission spectra in DCM (*E*_gap_ = 2.71 eV), we were able to obtain a LUMO energy value of −2.50 eV (Figure 4c).

The emission of **DICzTRZ** in degassed toluene is red-shifted at 477 nm compared to **ICzTRZ** [14], at 462 nm (Figure 4d). The excitation spectrum mirrors the profile of the UV–vis absorption (Supporting Information File 1, Figure S4a). Transient PL measurements in degassed toluene show mono-exponential prompt and delayed fluorescence decays at 8.94 ns and 28.83 µs, respectively (Supporting Information File 1, Figure S4c,d). After exposure to oxygen, the delayed fluorescence disappears while the prompt decay lifetime, τ_p_, is slightly reduced to 6.80 ns, implying the involvement of triplet states in the emission. When compared to **ICzTRZ** in degassed toluene, **DICzTRZ** presents comparable τ_p_ (9.0 ns for **ICzTRZ**), while we observe a substantial one order of magnitude decrease in the delayed lifetime, τ_d_, (229.2 µs for **ICzTRZ** [14]), reflective of a more efficient RISC process. **DICzTRZ** is less emissive than **ICzTRZ** (Φ_PL_ of 72% [14]), with Φ_PL_ in degassed toluene of 60% that decreases to 44% once exposed to oxygen. This reduction in Φ_PL_ is in part due to the decrease in the radiative decay rate given the smaller calculated oscillator strength for the emissive S_1_ state for this compound compared to **ICzTRZ**. The Δ*E*_ST_ of **DICzTRZ** in toluene glass at 77 K is 0.21 eV (see Supporting Information File 1, Figure S4, which is significantly smaller than the 0.32 eV obtained for **ICzTRZ** under the same conditions. The T_1_ levels of both **DICzTRZ** and **ICzTRZ** are comparable at 2.59 eV and 2.62 eV, respectively, while the S_1_ level for **DICzTRZ** is more stabilized at 2.80 eV vs 2.94 eV for **ICzTRZ**). We can clearly observe that the phosphorescence spectrum presents a well-defined structure, typical for transitions coming from a local excited (LE) type state on the diindolocarbazole. TDA-DFT calculations in the gas phase predict that the T_1_ state is CT in nature while the lowest-lying triplet states with LE character are T_3_ and T_4_ (T_3_ and T_4_ are at 2.73 eV and 2.75 eV, respectively, while T_1_ is at 2.64 eV, see Supporting Information File 1, Table S1 and Figure S3). The character of the different transitions was also evaluated by analysis of the natural transition orbitals (NTOs) (see Supporting Information File 1, Table S2). The T_1_ and T_2_ HONTO and LUNTO (highest occupied and lowest unoccupied natural Transition orbitals) are localized on the central diindolocarbazole and adjacent triazine, respectively, showing a clear CT between donor and acceptor moieties in the molecule. As previously mentioned, T_3_ and T_4_ present LE character, with the NTOs localized mainly the central di-indolocarbazole. The character of each of T_5_ and T_6_ is more difficult to assign as the electron density of the transition is localized on one of the indolocarbazole-triazine fragments and showing a high degree of overlap between the HONTO and LUNTO, which indicates a transition with a mixed CT and LE character. S_1_ also presents a clear CT transition from the diindolocarbazole to the triazine.

**Figure 5:**
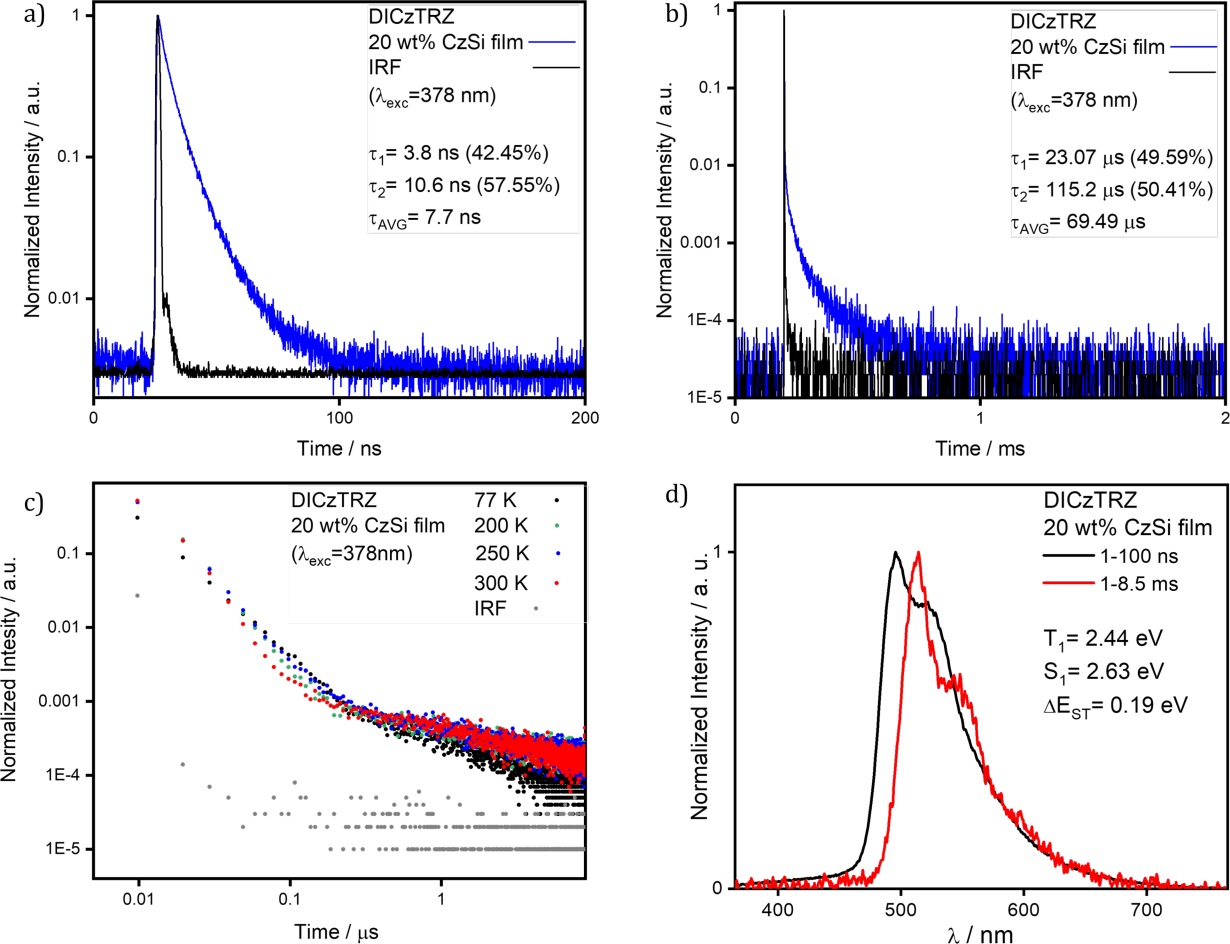
a) Prompt and b) delayed time-resolved decay in spin-coated 20 wt % CzSi film of **DICzTRZ** (λ_exc_ = 378 nm); c) delayed fluorescence decay data measured at different temperatures in spin-coated 20 wt % CzSi film of **DICzTRZ** (λ_exc_ = 378 nm); d) prompt fluorescence and phosphorescence spectra at 77 K in drop-casted 20 wt % CzSi film (λ_exc_ = 343 nm, prompt and delayed fluorescence spectra were obtained in the 1–100 ns and 1–10 ms time range, respectively).

With a view to incorporating **DICzTRZ** as the emitter in a solution-processed OLED, we next investigated the photophysical behavior of this compound in solid host matrices. We began with 10 wt % doped film of **DICzTRZ** in PMMA as the polarity of PMMA emulates well that of toluene [36]. The emission maximum in PMMA is 514 nm (Supporting Information File 1, Figure S5a) with a corresponding Φ_PL_ of 29% under N_2_. The significantly red-shifted emission in the PMMA film compared to that in toluene coupled with a significantly lower Φ_PL_ is suggestive that aggregation-caused quenching is prevalent in this host matrix. Transient PL measurement (Supporting Information File 1, Figure S5b,c) showed multiexponential decay kinetics and lifetimes with an average τ_p_ of 8.6 ns [τ_1_ = 3.5 ns (37.5%), τ_2_ = 11.6 ns (62.5%)] and an average τ_d_ of 156.1 µs [τ_1_ = 27.98 µs (39.5%), τ_2_ = 239.7 µs (60.5%)], respectively. The average prompt fluorescence lifetimes are of a similar magnitude to that of **ICzTRZ** (τ_p_ = 11.5 ns) [14] while the average delayed fluorescence decays much faster for **DICzTRZ** (τ_d_ = 252.8 µs for **ICzTRZ**) [14]. We next focused on the photophysical study in a suitably high triplet energy small molecule host material, CzSi (9-(4-*tert*-butylphenyl)-3,6-bis(triphenylsilyl)-9*H*-carbazole). The emission in CzSi at 488 nm, is only slightly red-shifted compared to that in toluene (Figure 4d). Gratifyingly, the Φ_PL_ is substantially higher at 57% in 20 wt % doped CzSi film, compared to that in the 10 wt % PMMA films (Table 1). In this host, transient PL measurements show the presence of both prompt and delayed fluorescence (Figure 5a,b) with respective average lifetimes of τ_p_ of 7.7 ns [τ_1_ = 3.8 ns (42.5%), τ_2_ = 10.6 ns (57.6%)] and τ_d_ of 69.49 µs [τ_1_ = 23.07 µs (49.6%), τ_2_ = 115.2 µs (50.4%)]. While the Φ_PL_ largely benefits from the change in the host, the lifetimes of the prompt fluorescence remain largely unchanged while we observe a much shorter delayed fluorescence. Both prompt and delayed lifetimes of **DICzTRZ** in CzSi are shorter than those of **ICzTRZ** in the same host (τ_p_ 9.5 ns, τ_d_ of 147.3 µs, Supporting Information File 1, Figure S5f,g). The Δ*E*_ST_ values in CzSi (Figure 5d) and PMMA (Supporting Information File 1, Figure S5d), are 0.19 eV and 0.03 eV, respectively. From a cross-comparison of the state energies (Table 1) we can see that the T_1_ state remains essentially the same regardless of the environment, this due to the LE nature of this excited state. The energy of the S_1_ state varies with the environments (with energies of 2.94 eV, 2.72 eV, and 2.75 eV for toluene solution, CzSi film and PMMA film, respectively for **ICzTRZ**), characteristic of a CT type state, but the shape of the spectra in all media adopt a structured profile, typical for LE-type states, suggesting a state of mixed CT and LE character (Supporting Information File 1, Figures S4d and S5d). **DICzTRZ** and **ICzTRZ** possess comparable Δ*E*_ST_ in CzSi, at 0.19 eV and 0.16 eV respectively. Temperature-dependent time-resolved PL decays (Figure 5c) reveal the clear increase in the intensity of the delayed emission with higher temperature, a hallmark of TADF.

**Table 1:** Photophysical properties of **ICzTRZ**[15] and **DICzTRZ**.

Material	Environment	λ_PL_ / nm^a^	Φ_PL_ N_2_ (air)^b^ / %	τ_p_, τ_d_^c^ / ns; μs	S_1_^d^ / eV	T_1_^e^ / eV	Δ*E*_ST_^f^ / eV

**ICzTRZ** ^g^	toluene (10^−5^ M)[15]	462	72 (56)^h^	9.0; 229.2	2.94	2.62	0.32
	CzSi 20 wt %^i^	475	63 (50)^j^	9.5; 147.3	2.72	2.56	0.16
	PMMA 10 wt %^i^ [15]	470	31 (28)^j^	115; 252.8	2.75	2.64	0.11

**DICzTRZ** ^k^	toluene (10^−5^ M)	477	60 (44)^h^	8.9; 28.83	2.80	2.59	0.21
	CzSi 20 wt %^i^	488	57 (42)^j^	7.7; 69.49	2.63	2.44	0.19
	PMMA 10 wt %^i^	514	29 (22)^j^	8.6; 156.1	2.61	2.58	0.03

^a^Measured at room temperature; ^b^λ_exc_ = 340 nm; ^c^τ_p_ (prompt lifetime) and *τ*_d_ (delayed lifetime) were obtained from the transient PL decay of degassed solution/doped film, λ_exc_ = 378 nm; ^d^S_1_ was obtained from the onset of the prompt emission measured at 77 K; ^e^T_1_ was obtained from the onset of the phosphorescence spectrum measured at 77 K; *^f^*Δ*E*_ST_ = S_1_ – T_1_. ^g^previous work [14]; ^h^obtained via the optically dilute method [37] (see Supporting Information File 1), quinine sulfate (0.5 M) in H_2_SO_4_ (aq) was used as the reference, Φ_PL_: 54.6% [38], λ_exc_ = 360 nm; ^i^spin-coated films; ^j^obtained via integrating sphere; ^k^this work.

In our previous work, we investigated the orientation of **ICzTRZ** in a variety of guest–host systems prepared by co-evaporation [13–14]. In all of these systems **ICzTRZ** presented nearly-completely horizontal orientation with anisotropy values in the range 0.06 to 0.12, depending on the host materials. However, in going from vacuum deposition, which was possible for the low-molecular weight emitter **ICzTRZ**, toward solution processing required for the much bigger **DICzTRZ**, one can expect significant changes of the orientation behaviour. It was shown, for example that phosphorescent iridium complexes like Ir(ppy)_2_(acac) display horizontal orientation (*a* ≅ 0.25) after vacuum co-evaporation, while the orientation changed toward isotropic in spin-coated films with PMMA as the host [39]. Moreover, upon solution processing with a low-*T*_g_ host like CBP, which is prone to crystallization, the obtained emitter orientation even turned vertical with the *a* factor approaching 0.40 [39].

**Figure 6:**
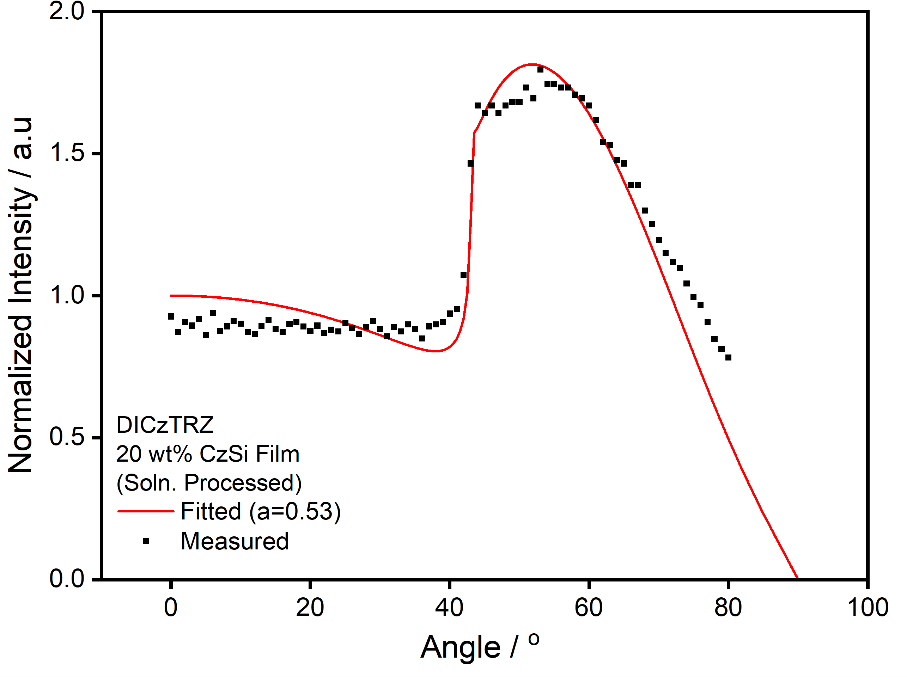
Angle-resolved photoluminescence measurement of a solution-processed film of 20 wt % **DICzTRZ** in CzSi. The red line shows a fit using the dipole emission model as described in detail in Supporting Information File 1, yielding an anisotropy factor, *a*, of 0.53 (data taken at λ_em_ = 500 nm).

Thus, measurements of the anisotropy factor were accordingly carried out for both **ICzTRZ** and **DICzTRZ**. Polarization and angle dependent luminescence spectroscopy was used to measure *a* for solution processed films of 20 wt % **DICzTRZ** in CzSi. The data were then analyzed via optical simulation to yield and anisotropy factor of 0.53, which disappointingly implies that the emitter presents a strongly vertical orientation (Figure 6); the corresponding measurement of 20 wt % **ICzTRZ** in CzSi is shown in Supporting Information File 1, Figure S6. It also shows vertical emitter orientation (*a* = 0.50; see fit in Supporting Information File 1, Figure S6).

Clearly, both emitters exhibit unfavourable orientation of their TDMs when processed from solution. As stated above, this change of orientation in relation to the used processing conditions is not unexpected and confirms – once more – that an important driving force for non-isotropic emitter orientation upon vacuum deposition is the non-equilibrium situation at the surface of a growing film, as suggested by the Ediger group [40]. This is not the case for solution processing where molecules in the liquid film can almost freely rotate and adopt a more or less random orientation before the solvent evaporates and their orientation is fixed in the solid film. There may also be some effect of the host on the resulting orientation as well, which seems to be the case here for CzSi where we observe pronounced vertical orientation of both emitters. There is a difficulty in designing host molecules that lead emitters to orient horizontally without sacrificing other preferable properties of the host; for example, high triplet energy, good film-forming ability.

### OLED devices

**Figure 7:**
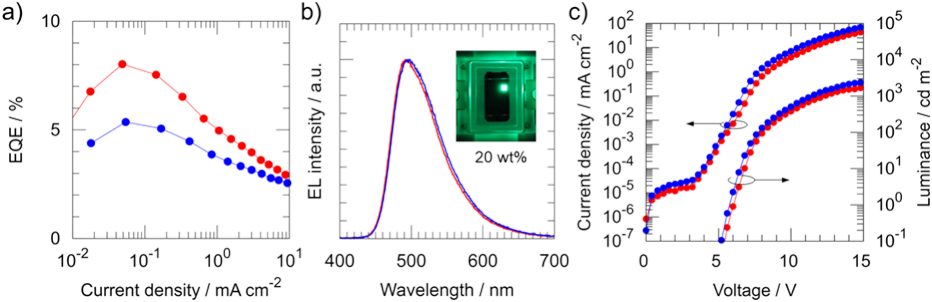
Device characteristics of 20 and 30 wt % **DICzTRZ**-based OLEDs, which are represented by red and blue, respectively. a) EQE-current density, b) EL spectra and c) current density-voltage-luminance properties.

Finally, **DICzTRZ** and **ICzTRZ**-based OLEDs were fabricated using the following device structure: ITO (indium tin oxide) (50 nm)/PEDOT:PSS (poly(3,4-ethylenedioxythiophene) polystyrene sulfonate) (35 nm)/PVK (poly(9-vinylcarbazole)) (10 nm)/*X* wt % **DICzTRZ** or **ICzTRZ**: CzSi (20 nm)/PPF (2,8-bis(diphenylphosphoryl)dibenzo[*b*,*d*]furan) (5 nm)/TPBi (1,3,5-tris(1-phenyl-1*H*-benzo[*d*]imidazol-2-yl)benzene) (50 nm)/Liq (lithium quinolin-8-olate) (1 nm)/Al (80 nm), where *X* is 20 or 30. The PVK layer is applied to facilitate hole injection from PEDOT:PSS to the emitting layer. Besides, PVK and PPF, possessing high T_1_ energies of 3.0 eV [41] and 3.1 eV [42], respectively, were inserted to confine the excitons in the emitting layer. PEDOT:PSS, PVK and the emitting layer were fabricated by spin-coating, and the other layers were vacuum-deposited. Device characteristics are shown in Figure 7 for **DICzTRZ**, Figure S7 (Supporting Information File 1) for **ICzTRZ**, and the device performance is summarized in Table 2. As shown in Table 2, 20 wt % **DICzTRZ**-based OLEDs achieved EQE_max_ of 8.4% and λ_EL_ of 494 nm with CIE coordinates (*x*, *y*) of (0.22, 0.47) at 1 mA cm^−2^. The 20 wt % **ICzTRZ**-based OLEDs exhibited a slightly higher EQE_max_ of 11.6% and blue-shifted emission with λ_EL_ of 485 nm. This result is consistent with that of the photophysical measurements for 20 wt % TADF emitter:CzSi films (Φ_PL_ = 57% and λ_PL_ = 488 nm for **DICzTRZ**, Φ_PL_ = 63% and λ_PL_ = 475 nm for **ICzTRZ**, respectively).

**Table 2:** Device performances of *X* wt % **ICzTRZ**- and **DICzTRZ**-based OLEDs (where *X* = 20, 30).

Emitter	Concentration / %	EQE_max_ / %	λ_EL_ / nm^a^	CIE (*x*, *y*)

**ICzTRZ**	20	11.6	485	(0.19, 0.37)
	30	6.6	485	(0.20, 0.39)

**DICzTRZ**	20	8.4	494	(0.22, 0.47)
	30	5.4	498	(0.22, 0.49)

^a^Determined from EL spectrum at 1 mA cm^−2^.

**Figure 8:**
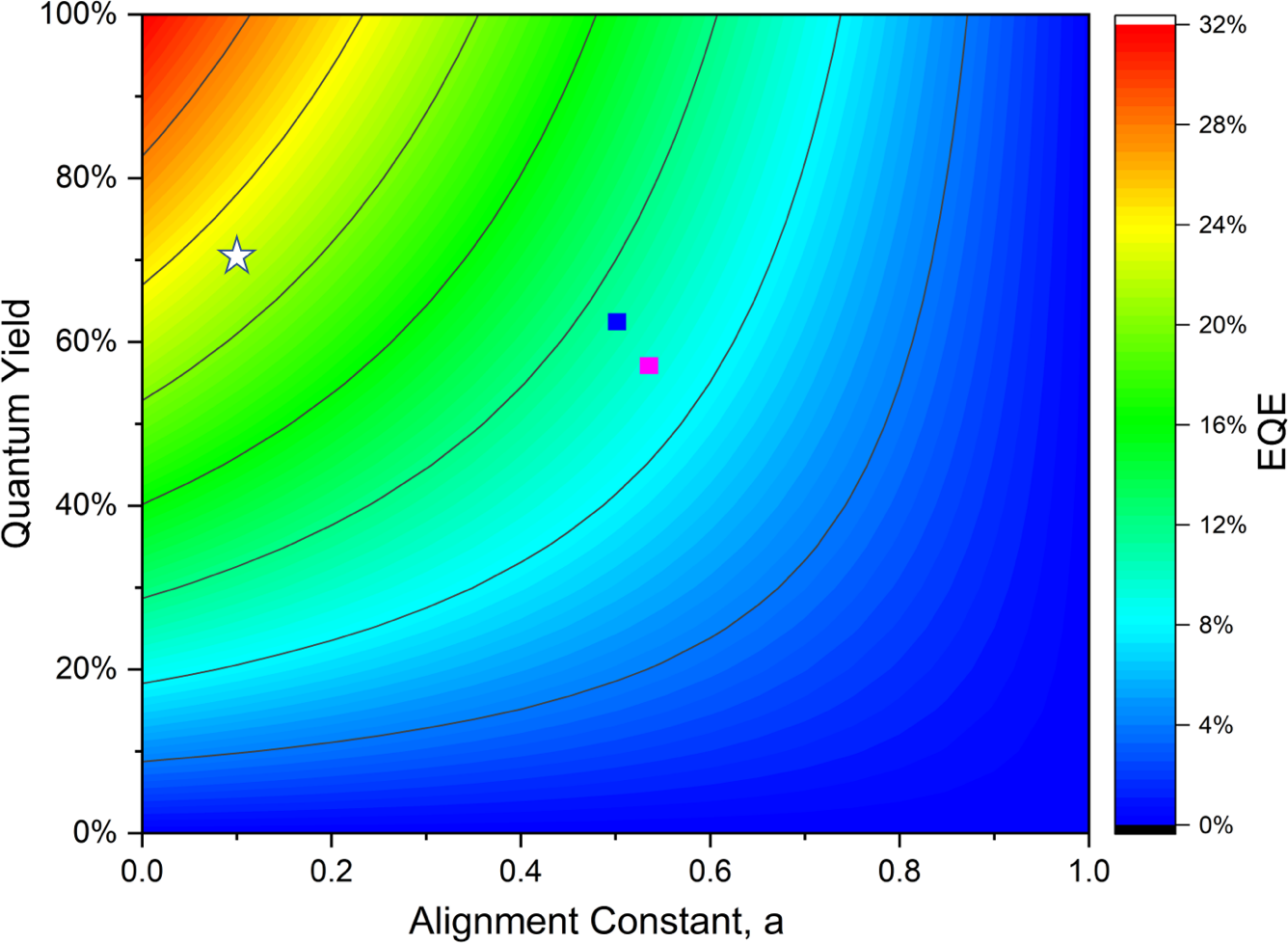
Device efficiency simulation of the fabricated OLEDs depicting the variation in EQE with varied PL quantum yield (vertical axis) and anisotropy factor (horizontal axis). The predicted EQEs are indicated with pink and blue rectangular marks for the **DICzTRZ** and **ICzTRZ** solution-processed OLEDs, respectively. The white star shows the predicted EQE for an evaporated **ICzTRZ** OLED with the orientation and PLQY taken from ref. [14]. All of the simulated EQEs agree fairly well with the experimental results.

We next simulated the device EQE (Supporting Information File 1, Figure S8). As shown in Figure 8, with the pre-determined parameters (Φ_PL_ and *a*) along with the optical constants of the different materials in the OLED stack, we predict the **DICzTRZ** device to show an EQE_max_ of between 9–10%, which aligns well with the measured EQE_max_, whereas the corresponding solution-processed **ICzTRZ** OLED (see Supporting Information File 1, Figure S7 for experimental data) is expected to show an EQE_max_ of about 11%, again in fairly good agreement with the measured results. The simulation also demonstrates that for **ICzTRZ** with the typically obtained alignment factor of about 0.1 in an evaporated device (and a slightly higher Φ_PL_ of 70% as documented in ref. [14], a device EQE of about 22% can be expected as a result of the horizontal orientation of the emitter within an evaporated EML. Thus, vacuum deposition of this family of TADF emitters is clearly superior to solution processing.

## Conclusion

Building upon our previously reported emitter, **ICzTRZ**, here we presented a dual emitter strategy consisting of two **ICzTRZ** moieties covalently linked together in the form of **DICzTRZ**. DFT calculations showed a much larger density of triplet states, which suggests that RISC will be faster in this compound compared to its parent. The twin design strategy leads to an enhancement in the molar extinction coefficient of the low-lying CT states, accompanied by a red-shift in the emission. The 20 wt % doped CzSi film of **DICzTRZ** emits in the blue at 488 nm and shows a photoluminescence quantum yield of 57.1%. The Φ_PL_ of **DICzTRZ** is slightly lower than that of **ICzTRZ** (63% under N_2_ [14]), in line with its lower computed oscillator strength. **DICzTRZ** shows both prompt and delayed fluorescence, with a τ_p_ that remains largely unchanged from that of **ICzTRZ**, while its τ_d_ is significantly shorter. Unfortunately, the TDM of this material is not preferentially horizontally oriented in the solution-processed film, which is not unexpected in solution-processed films. The combination of its lower Φ_PL_ and the vertical orientation of its TDM are the primary factors governing the relatively poorer device performance, with an EQE_max_ of 8.4%, compared to the vacuum-deposited OLED with **ICzTRZ** [14].

## Supporting Information

The research data supporting this publication can be accessed at doi:10.17630/4a01d3e3-71bc-4ebb-9812-c4b838e13573.

File 1 Synthesis protocols, NMR spectra, supplementary photophysical measurements, computational data obtained from DFT and TD-DFT and electroluminescence data.

File 2 *xyz* Coordinates corresponding to the ground state optimized geometry of **DICzTRZ**.
