# Supplementary material for: Effect of a twin-emitter design strategy on a previously reported thermally activated delayed fluorescence organic light-emitting diode
Source: Beilstein J Org Chem. 2021 Dec 8;17:2894–905. doi: 10.3762/bjoc.17.197 (PMC8685574; doi:10.3762/bjoc.17.197)
Supplement: File 1 — Synthesis protocols, NMR spectra, supplementary photophysical measurements, computational data obtained from DFT and TD-DFT and electroluminescence data. [file Beilstein_J_Org_Chem-17-2894-s001.pdf]

BEILSTEIN JOURNAL OF ORGANIC CHEMISTRY

Supporting Information

for

Effect of a twin-emitter design strategy on a previously reported thermally activated delayed fluorescence organic light-emitting diode

Ettore Crovini, Zhen Zhang, Yu Kusakabe, Yongxia Ren, Yoshimasa Wada, Bilal A. Naqvi, Prakhar Sahay, Tomas Matulaitis, Stefan Diesing, Ifor D. W. Samuel, Wolfgang Brütting, Katsuaki Suzuki, Hironori Kaji, Stefan Bräse and Eli Zysman-Colman

Beilstein J. Org. Chem. **2021,** *17,* 2894–2905. doi:10.3762/bjoc.17.197

Synthesis protocols, NMR spectra, supplementary photophysical measurements, computational data obtained from DFT and TD-DFT and electroluminescence data

License and Terms: This is a supporting information file under the terms of the Creative Commons Attribution License (https://creativecommons.org/licenses/by/4.0). Please note that the reuse, redistribution and reproduction in particular requires that the author(s) and source are credited and that individual graphics may be subject to special legal provisions.
The license is subject to the *Beilstein Journal of Organic Chemistry* terms and conditions: (https://www.beilstein-journals.org/bjoc/terms)

SUPPORTING INFORMATION

General methods..........S2

Experimental section..........S7

DFT calculations..........S9

Optoelectronic characterization..........S14

Orientation measurements..........S17

Device simulation..........S19

References..........S20

General methods

NMR spectra were recorded using the following devices: ^{1}H NMR: Bruker Avance 400 (400 MHz), ^{13}C NMR: Bruker AM 400 (100 MHz). Chloroform-d_1 from Eurisotop was used as solvent for NMR. Chemical shifts δ were expressed in parts per million (ppm) and referenced to chloroform (^{1}H: δ = 7.26 ppm, ^{13}C: δ = 77.16 ppm). The signal structure is described as follows: s = singlet, d = doublet, t = triplet, q = quartet, quin = quintet, b = broad singlet, m = multiplet, dd = doublet of doublet, dt = doublet of triplet. The spectra were analysed according to first order. All coupling constants are absolute values and expressed in Hertz (Hz).

Matrix-assisted laser desorption ionization-time of flight (MALDI-TOF) mass spectra were recorded on a BRUKER Biflex IV spectrometer with a pulsed ultraviolet nitrogen laser (200 µJ at 337 nm) and a time-of-flight mass analyzer with a 125 cm linear flight path.

The infrared spectra of solid samples were recorded on Bruker IFS 88 and measured by attenuated total reflection (ATR method). Absorption is given in wave numbers ῡ [cm^{-1}].

Analytical thin layer chromatography (TLC) was carried out on Merck silica gel coated aluminum plates (silica gel 60, F254), detected under UV-light at 254 nm or stained with "Seebach staining solution" (mixture of molybdato phosphoric acid, cerium(IV) sulfate tetrahydrate, sulfuric acid and water) or basic potassium permanganate solution. Solvent mixtures are understood as volume/volume. Spectroscopic grade solvents were either purchased from Fisher Scientific or Sigma Aldrich (<50 ppm H_2O). Other solvents were acquired from Sigma Aldrich, Carl Roth or Fisher Scientific. Unless otherwise stated, all solvents and reagents were used without further purification.

Electrochemistry measurements. (In a manner analogous to [1]) Cyclic Voltammetry (CV) and Differential Pulse Voltammetry analyses were performed on an Electrochemical Analyzer potentiostat model 620D from CH Instruments. Sample of **DICzTRZ** were prepared in DCM that was degassed by sparging with DCM-saturated nitrogen gas for 10 minutes before measurements. All measurements were performed in 0.1 M DCM solution of tetrabutylammonium hexafluorophosphate, which was used as the supporting electrolyte. An Ag/Ag^{+} electrode was used as the reference electrode while a glassy carbon electrode and a platinum wire were used as the working electrode and counter electrode, respectively. The redox potentials are reported relative to a saturated calomel electrode (SCE) with a

ferrocene/ferrocenium (Fc/Fc^+) redox couple as the internal standard (0.46 V vs SCE for DCM [2]).

Photophysical measurements. (In a manner analogous to [1]) Optically dilute solutions of concentrations on the order of 10^{-5} M were prepared in HPLC grade methyl-cyclohexane, toluene, DCM, THF, and MeCN for absorption and emission analysis. Absorption spectra were recorded at room temperature on a Shimadzu UV-1800 double beam spectrophotometer with a 1 cm quartz cuvette. Molar absorptivity determination was verified by linear least-squares fit of values obtained from at least five independent solutions at varying concentrations with absorbance ranging from 1.66×10^{-5} to 1.93×10^{-6} M.

For emission studies, aerated solutions were bubbled with compressed air for 5 minutes and spectra were taken using the same cuvette as for the absorption analysis. Degassed solutions were prepared via three freeze-pump-thaw cycles and spectra were taken using a home-made Schlenk quartz cuvette. Steady-state emission, excitation spectra and time-resolved emission spectra were recorded at 298 K using an Edinburgh Instruments F980. Samples were excited at 340 nm for steady-state measurements and at 378 nm for time-resolved measurements. Photoluminescence quantum yields for solutions were determined using the optically dilute method [3] in which four sample solutions with absorbances of ca. 0.104, 0.087, 0.065 and 0.047, at 360 nm were used. The Beer–Lambert law was found to remain linear at the concentrations of the solutions. For each sample, linearity between absorption and emission intensity was verified through linear regression analysis with the Pearson regression factor (R^2) for the linear fit of the data set surpassing 0.9. Individual relative quantum yield values were calculated for each solution and the values reported represent the slope obtained from the linear fit of these results. The equation $\Phi_s = \Phi_r(A_r/A_s)(I_s/I_r)(n_s/n_r)^2$ was used to calculate the relative quantum yield of the sample, where (Φ_r) is the absolute quantum yield of the external reference quinine sulfate (Φ_r = 54.6% in 1 N H_2SO_4) [4], A stands for the absorbance at the excitation wavelength, I is the integrated area under the corrected emission curve and n is the refractive index of the solvent. The subscripts “s” and “r” representing sample and reference, respectively. The experimental uncertainty in the emission quantum yields is conservatively estimated to be 10%, though we have found that statistically, we can reproduce Φ_{PL} values to 3% relative error. Thin-film Φ_{PL} measurements were performed using an integrating sphere in a Hamamatsu C9920-02 system. A xenon lamp coupled to a monochromator enabled excitation selectivity, chosen here to be 340 nm. The output was then fed into the integrating sphere via a fibre,

exciting the sample. PL spectra were collected with a multimode fibre and detected with a back-thinned CCD. Doped thin films were prepared by mixing sample (3 wt %) and host material in toluene solution, followed by spin-casting on a quartz substrate. The Φ_{PL} of the films were then measured in air and by purging the integrating sphere with flowing N_2 gas. Time-resolved PL measurements of the thin films were carried out using the time-correlated single-photon counting technique. The samples were excited at 378 nm by a pulsed laser diode (Picoquant, model PLS 370) and were kept in a vacuum of <8 × 10^{-4} mbar.

The singlet-triplet splitting energy, ΔE_{ST}, was estimated by recording the prompt fluorescence spectra and phosphorescence emission at 77 K. The films were excited either by a Q-switched Nd:YAG laser emitting at 343 nm (Laser-export) or by a femtosecond optical parametric amplifier emitting at 320 nm (Orpheus-N, Light Conversion). Emission from the samples was focused onto a spectrograph (Chromex imaging, 250is spectrograph) and detected on a sensitive gated iCCD camera (Stanford Computer Optics, 4Picos) having a sub-nanosecond resolution. Prompt fluorescence spectra were measured 1 ns after the excitation of the femtosecond laser with iCCD exposure time of 100 ns. Phosphorescence spectra were measured 1 ms after the excitation of the Nd:YAG laser with iCCD exposure time of 10 ms.

Fitting time-resolved luminescence measurements: Time-resolved PL measurements were fitted to a sum of exponentials decay model with chi-squared (χ^2) values between 1 and 2, using the EI FLS980 software. Each component of the decay is assigned a weight, (w_i), which is the contribution of the emission from each component to the total emission.

The average lifetime was then calculated using the following:

- Two exponential decay model:

$$\tau_{AVG} = \tau_1 w_1 + \tau_2 w_2$$

with weight defined as $w_1 = \frac{A1\tau_1}{A1\tau_1 + A2\tau_2}$ and $w_2 = \frac{A2\tau_2}{A1\tau_1 + A2\tau_2}$ where A1 and A2 are the preexponential-factors of each component.

Theoretical calculations: All ground state optimizations have been carried out at the Density Functional Theory (DFT) level with Gaussian09 [5] using the PBE0 [6] functional and the 6-31G(d,p) basis set [7]. Excited state calculations have been performed at Time-Dependent DFT (TD-DFT) within the Tamm-Dancoff approximation (TDA[8,9]) using the same functional and

basis set as for ground state geometry optimization. This methodology has been demonstrated to show a quantitative estimate of ΔE_{ST} in comparison to experiment [10].

Angular dependent photoluminescence spectroscopy. Thin films of emitter and host were spin-coated on a pre-cleaned quartz glass substrate. This substrate was then glued with an index matching fluid on a fused-silica prism which was mounted on the rotating stage. The organic film was then irradiated with a UV laser (Kimmon, HeCd laser, λ = 325 nm) under vertical incidence and was rotated from −90° to +90° with respect to the substrate normal. The luminescence was recorded with a grating spectrograph coupled to a liquid-nitrogen cooled charge-coupled device (Princeton Instruments Acton 2300i with PyLoN detector) in s and p polarization mode. P-polarized signal was then subjected to numerical simulation to calculate the orientation factor (a):

$$a = \frac{\Sigma p_z^2}{\Sigma p^2}$$

Where Σp_z^2 is the sum of the power emitted by vertically oriented dipoles and Σp^2 is the sum of the power emitted by all emitting dipoles [11]. The parameter a (or synonymously $\Theta_v = \langle\cos^2\vartheta\rangle$) denotes the second moment of the TDM's angular distribution around the surface normal of the film, where ϑ is the angle between the molecule's TDM vector and the said direction. The details about the method can be further found in the reference [11,12].

OLED fabrication and characterization: OLEDs were fabricated according to following procedure. Before fabrication, the ITO patterned glass substrates were cleaned by sonication in detergent and H_2O (twice) for 10 minutes, then by hot steam of 2-propanol for 5 minutes followed by UV-O_3 irradiation for 30 minutes. Subsequently, 50 wt % PEDOT:PSS (CLEVIOUS^{TM} P VP CH 8000, Heraeus) : ultrapure H_2O was spin-coated on cleaned ITO glass substrates at 4000 rpm for 12 s, dried at 150 °C for 10 minutes and cooled at room temperature (rt) with N_2 flow for 5 minutes. 10 mg mL^{-1} of PVK (Mw ≈ 1,100,000) in 1,2-dichlorobenzene solution was spin-coated onto the PEDOP:PSS layer at 2000 rpm for 30 s, dried at 120 ℃ for 10 minutes and cooled for at rt with N_2 flow for 5 minutes. 10 mg mL^{-1} of emitter in toluene solution was spin-coated on the PVK layer at 2200 rpm for 30 s, dried at 100 °C for 10 minutes

and cooled at rt with N_2 flow for 5 minutes. Subsequent layers were vacuum-deposited using deposition apparatus (SE-4260, ALS Technology, Japan) at $\approx 10^{-4}$ Pa. To remove both oxygen and moisture, OLEDs were sealed with glass caps containing getter films in a glove box. Device characterization was performed by an integrating sphere (Hamamatsu Photonics, C9920-12) with Keithley 2400 Source Meter. During this characterization, voltages incremented by 400 mV were applied to devices.

Experimental section

5,5',11,11'-Tetrakis(4-(4,6-bis(4-(*tert*-butyl)phenyl)-1,3,5-triazin-2-yl)phenyl)-5,5',11,11'-tetrahydro-2,2'-biindolo[3,2-*b*]carbazole (DICzTRZ)

A 50 mL round flask was charged with $FeCl_3$ (32.4 mg, 200 µmol, 3.73 equiv.) and DCM (4.0 mL) under argon. Then, 9,9'-bis(4-(4,6-bis(4-(*tert*-butyl)phenyl)-1,3,5-triazin-2-yl)phenyl)-9*H*,9'*H*-3,3'-bicarbazole (58.5 mg, 53.4 µmol, 1.00 equiv) in DCM (2.0 mL) was very slowly added. The mixture was stirred at 60 °C for 12 h. A solution of 10% aqueous sodium hydroxide solution (20 mL) was added into the mixture, the organic phase was extracted with DCM (3 × 30 mL). The organic layer was dried over $MgSO_4$ and the solvent was removed under reduced pressure. The obtained crude product was purified via column chromatography on silica gel (cyclohexane/dichloromethane = 2:1) to yield the title compound as a yellow luminescent solid (38.5 mg, 17.6 µmol, 66%).

Mp = decomposition above 400 °C – **R*f*** = 0.40 (cyclohexane/dichloromethane = 2:1). – **^{1}H NMR** (500 MHz, $CDCl_3$) δ = 9.09 (t, *J* = 8.3 Hz, 8H), 8.75 (d, *J* = 8.5 Hz, 8H), 8.70 (d, *J* = 8.3 Hz, 8H), 8.49 (s, 2H), 8.36 (s, 2H), 8.25 (s, 2H), 8.17 (d, *J* = 7.7 Hz, 2H), 7.96 (d, *J* = 8.2 Hz, 8H), 7.85 (d, *J* = 8.6 Hz, 2H), 7.72–7.50 (m, 20H), 7.50–7.42 (m, 2H), 7.35–7.27 (m, 2H), 1.44 (s, 36H), 1.33 (s, 36H) ppm. – **^{13}C NMR** (125 MHz, $CDCl_3$) δ = 171.6, 171.5, 170.7, 170.6, 156.1, 156.0, 142.1, 142.0, 141.4, 140.5, 137.0, 136.5, 134.7, 134.5, 133.6, 133.5, 130.7, 128.9, 128.8, 126.4, 126.2, 125.7, 125.6, 124.6, 124.0, 123.9, 120.5, 120.0, 119.2, 113.9, 110.0, 109.8, 100.4, 100.3, 35.2, 35.0, 31.3, 31.2 ppm. – **IR** (ATR, ṽ) = 2959 (w), 2932 (w), 2904 (w), 1605 (w), 1578 (m), 1503 (vs), 1476 (s), 1446 (vs), 1408 (s), 1367 (vs), 1356 (vs), 1322 (m), 1312 (m), 1264 (m), 1231 (m), 1190 (m), 1173 (m), 1150 (m), 1128 (w), 1106 (m), 1057 (w), 1016 (m), 928 (w), 856 (w), 849 (w), 833 (w), 815 (vs), 795 (m), 762 (w), 735 (m), 727 (w), 704 (m), 684 (w), 602 (w), 577 (m), 550 (s), 509 (w), 480 (w), 453 (w), 414 (w), 377 (w) cm^{-1}. – **MS** (MALDI-TOF), *m/z* (%): 2188 $[M]^+$.

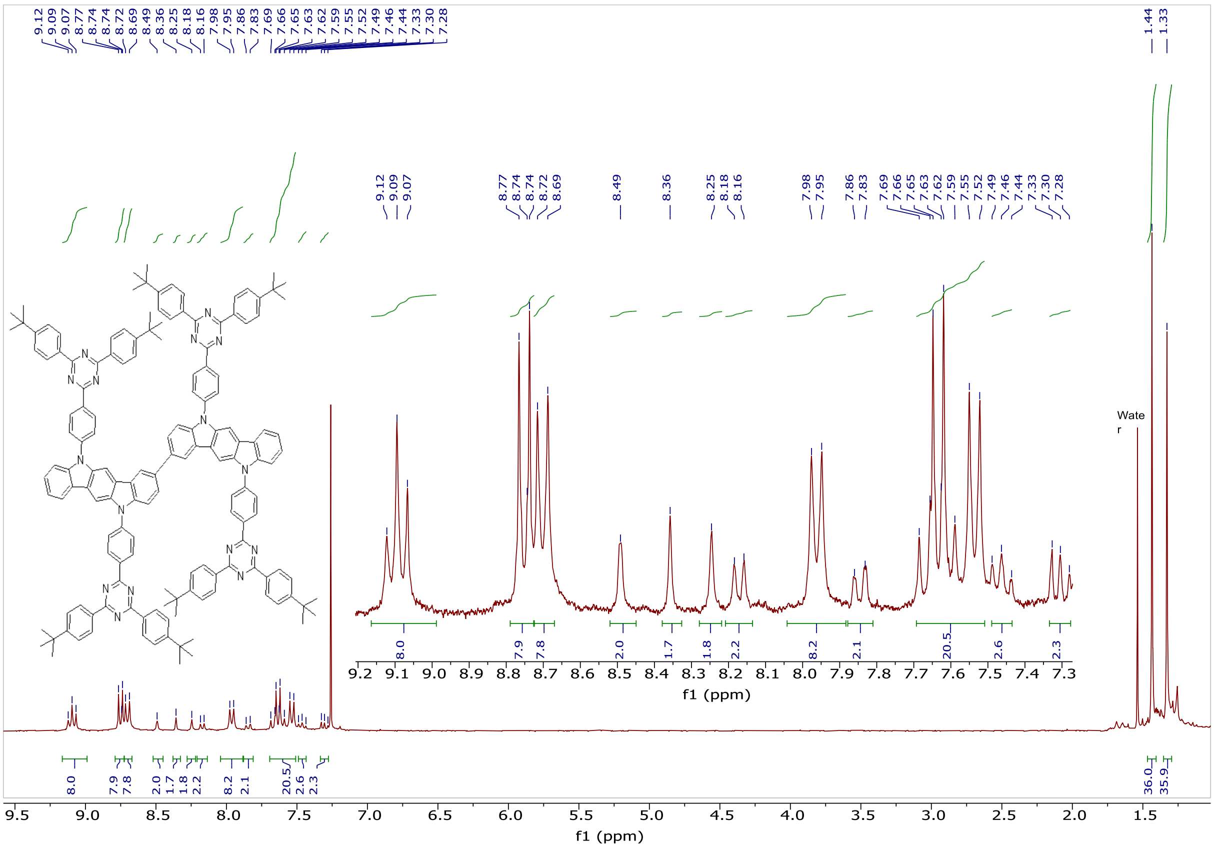

Figure S1. ^{1}H NMR of **DICzTRZ** in $CDCl_3$-*d*$_1$

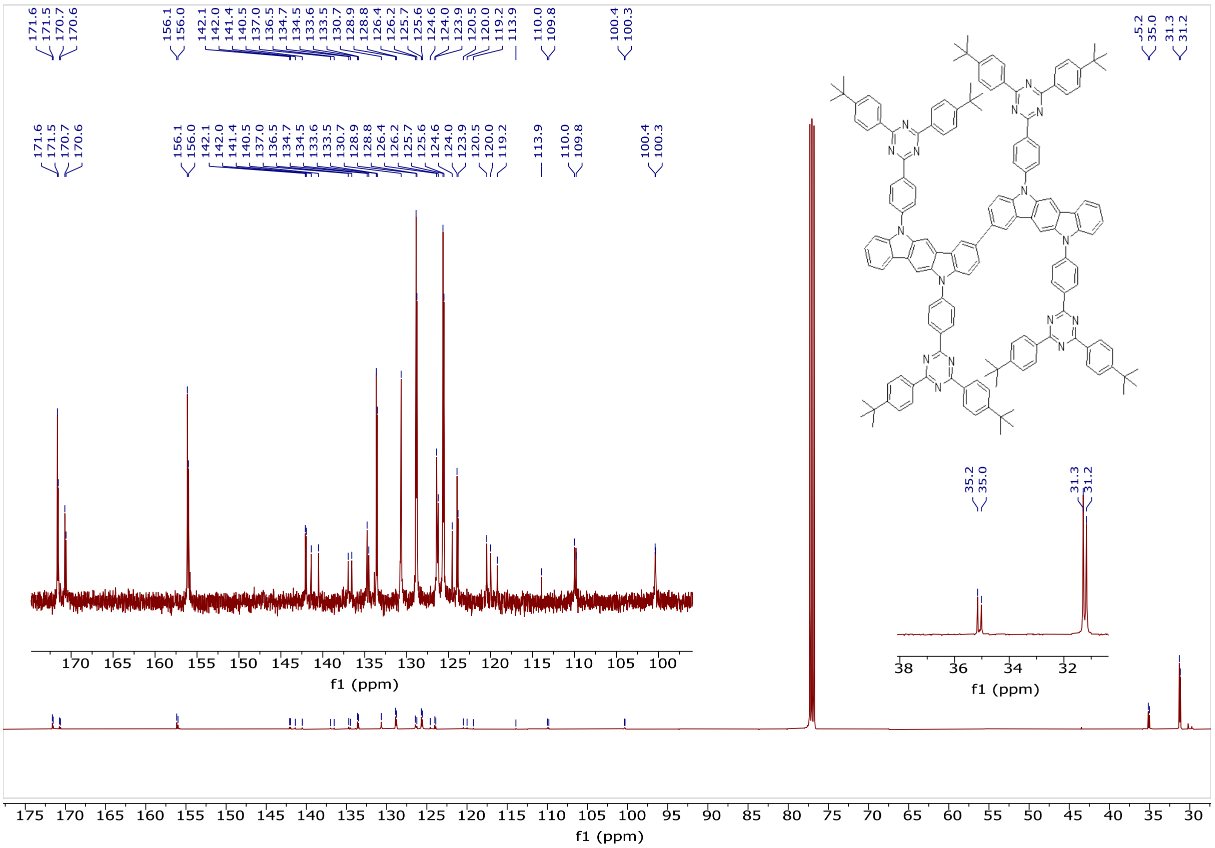

Figure S2. ^{13}C NMR of **DICzTRZ** in $CDCl_3$-*d*$_1$

DFT calculations

Table **S1.** Excited states properties of **DICzTRZ**

**Excited State**	**Energy / eV**	**Nature**	**Character of the transition**
T_1	2.64	**HOMO → LUMO (0.71)**<br>HOMO-5 → LUMO+1 (0.03)<br>HOMO → LUMO+10 (0.02)<br>HOMO-1 → LUMO+1 (0.12)	CT
T_2	2.67	**HOMO → LUMO+1 (0.67)**<br>HOMO-5 → LUMO (0.03)<br>HOMO-1 → LUMO+1 (0.03)<br>HOMO-1 → LUMO (0.16)	CT
T_3	2.73	**HOMO → LUMO+8 (0.61)**<br>HOMO-1 → LUMO+8 (0.06)<br>HOMO-1 → LUMO+9 (0.22)	LE (DICz)
T_4	2.75	**HOMO → LUMO+9 (0.43)**<br>HOMO-1 → LUMO+2 (0.02)<br>HOMO → LUMO+7 (0.02)<br>HOMO → LUMO+2 (0.13)<br>HOMO-1 → LUMO+8 (0.21)	LE (DICz)
T_5	2.76	**HOMO → LUMO+2 (0.38)**<br>HOMO → LUMO+9 (0.13)<br>HOMO-1 → LUMO+6 (0.03)<br>HOMO-1 → LUMO+5 (0.02)<br>HOMO-4 → LUMO+2 (0.02)<br>HOMO → LUMO+5 (0.02)<br>HOMO-1 → LUMO+8 (0.05)<br>HOMO → LUMO+6 (0.06)<br>HOMO-1 → LUMO+2 (0.10)	CT
T_6	2.82	HOMO → LUMO+5 (0.50)<br>**HOMO-1 → LUMO+5 (0.11)**<br>HOMO-1 → LUMO+2 (0.09)<br>HOMO → LUMO+2 (0.05)<br>HOMO-4 → LUMO+5 (0.04)	CT
S1 (f=0.62)	2.83	**HOMO → LUMO (0.88)**<br>HOMO-1 → LUMO+1 (0.07)	CT

a) HOMO -1

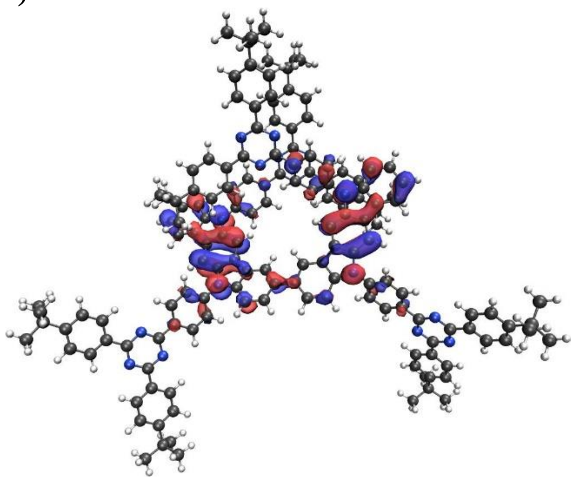

b) HOMO

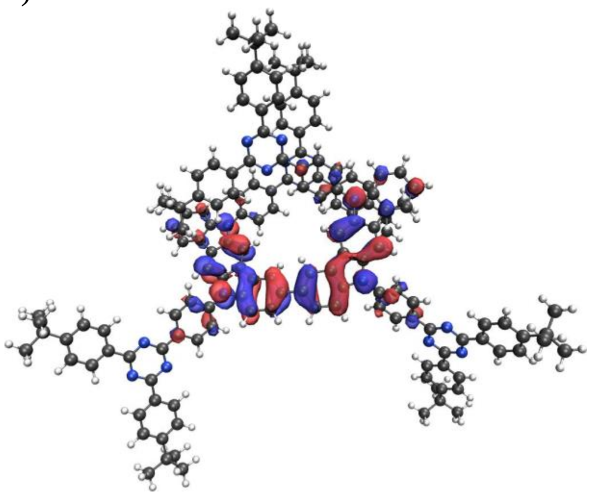

c) LUMO

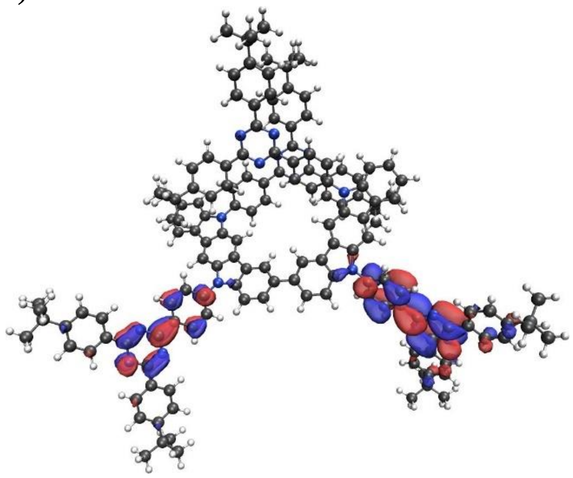

d) LUMO +1

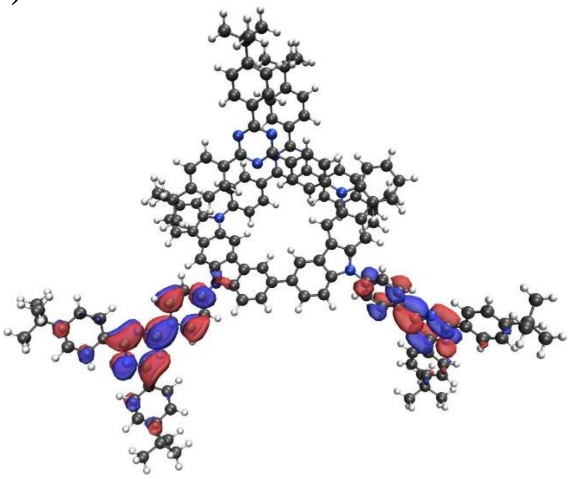

e) LUMO +2

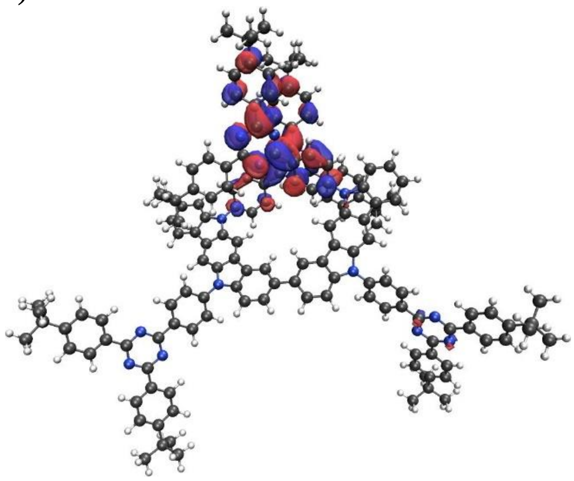

f) LUMO +5

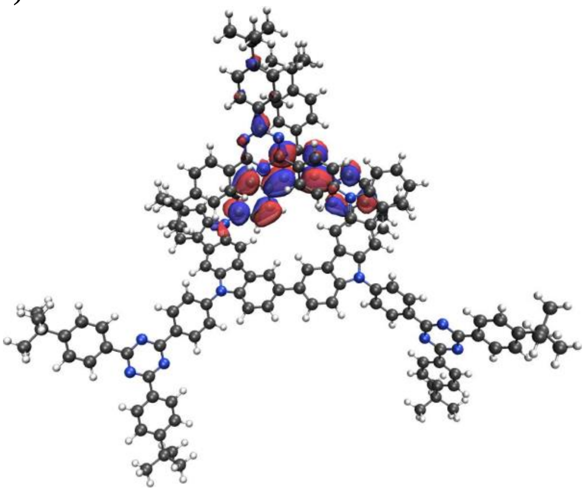

g) LUMO +8

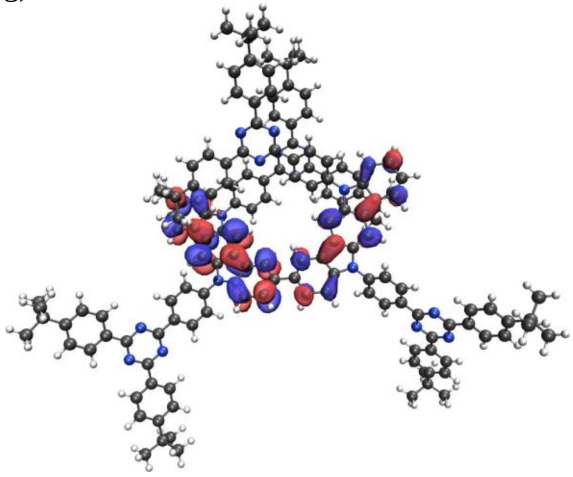

h) LUMO +9

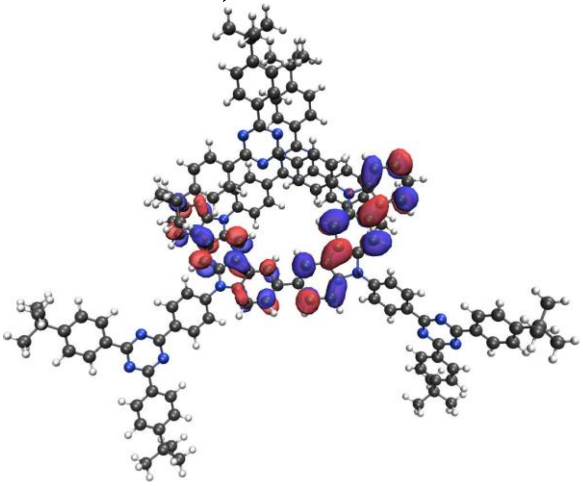

Figure **S3.** Electronic density surfaces of a) HOMO -1, b) HOMO, c) LUMO, d) LUMO +1, e) LUMO +2, f) LUMO +5, g) LUMO +8, h) LUMO +9 (Isovalue for new surfaces: MO = 0.02, Density = 0.0004).

Table **S2.** Electronic density surfaces of NOTOs of **DICzTRZ** (Isovalue for new surfaces: MO = 0.02, Density = 0.0004).

State	HONTO	LUNTO	Character
T_1	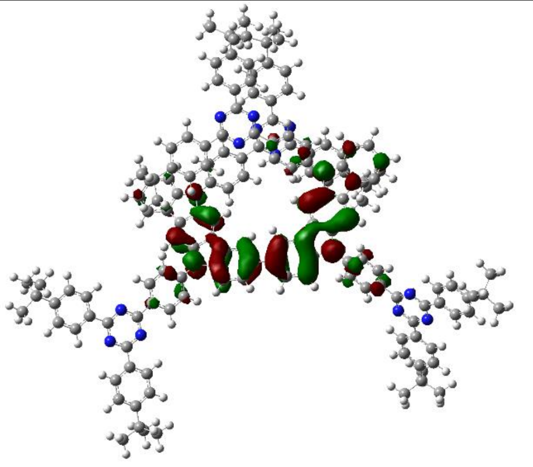	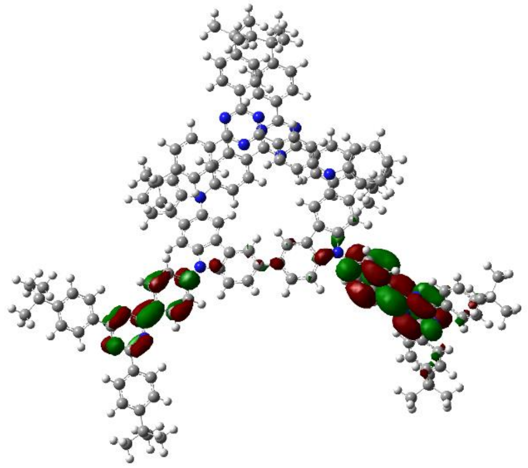	CT
T_2	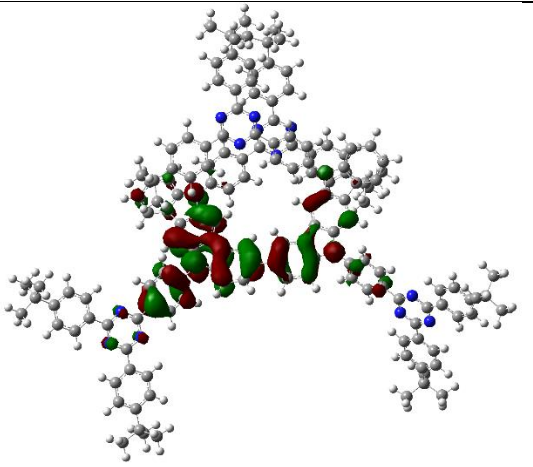	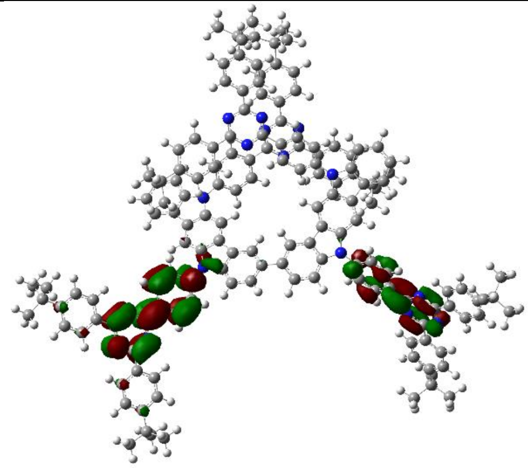	CT

T_3	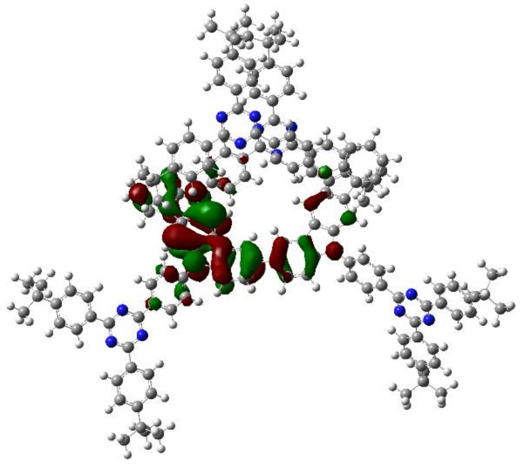	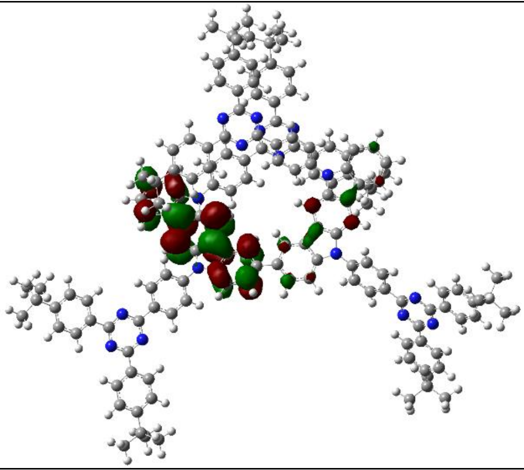	LE
T_4	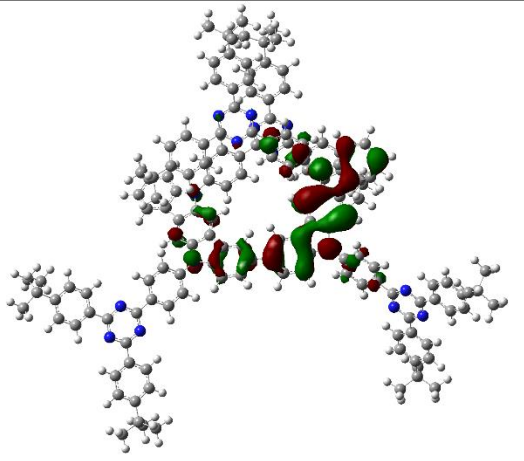	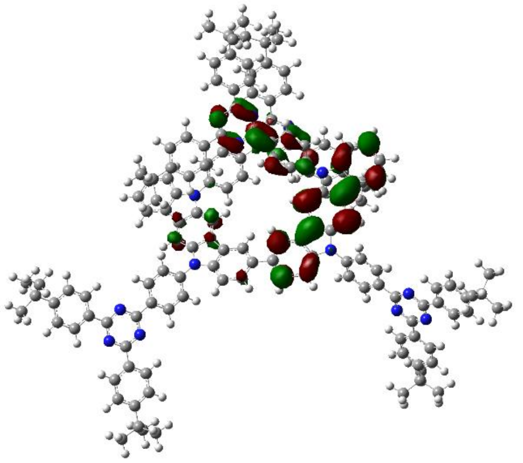	LE
T_5	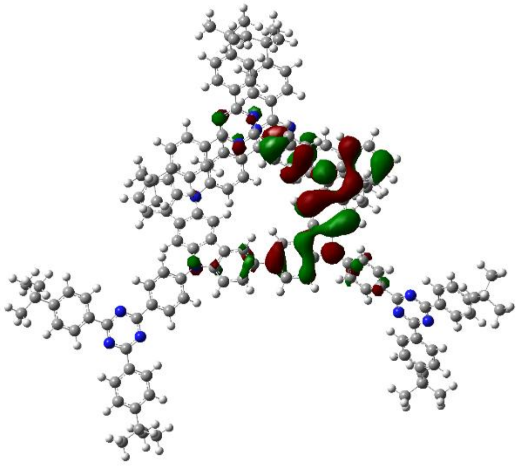	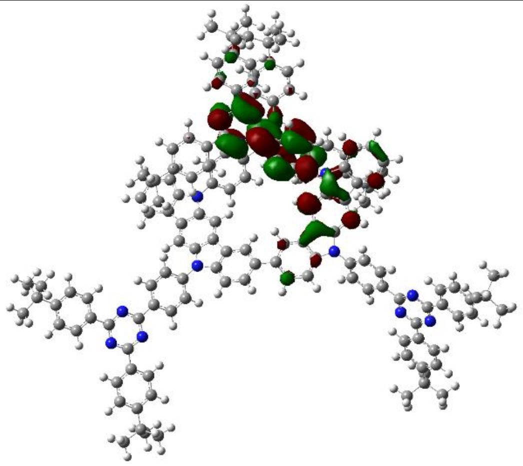	Mixed LE-CT
T_6	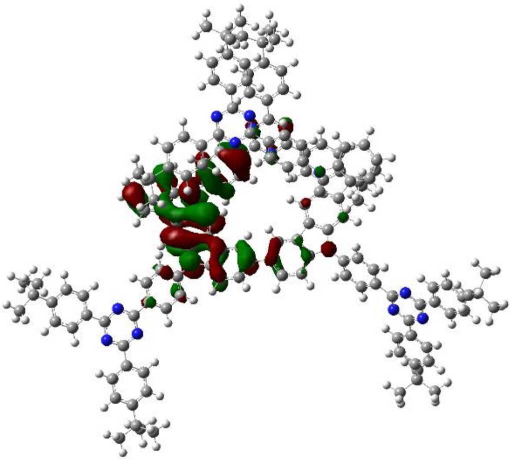	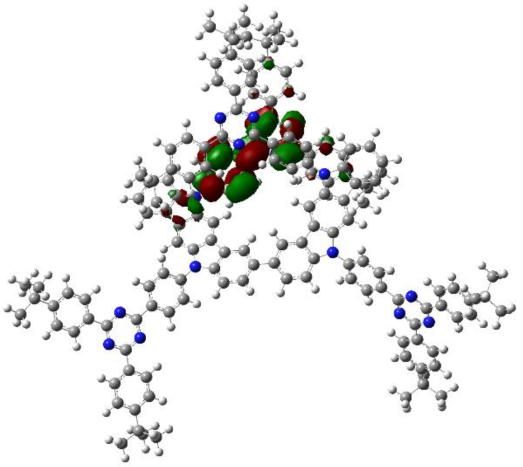	Mixed LE-CT

S_1	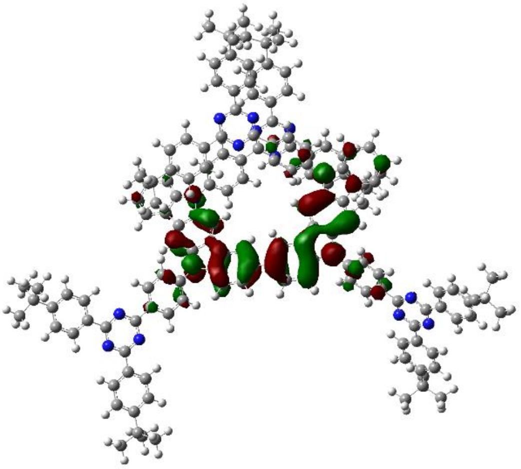	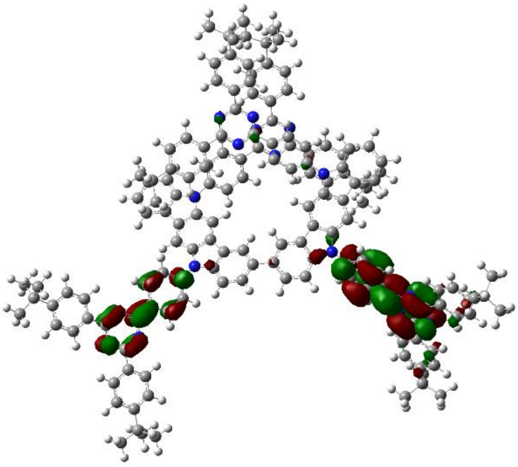	CT

Optoelectronic Characterization

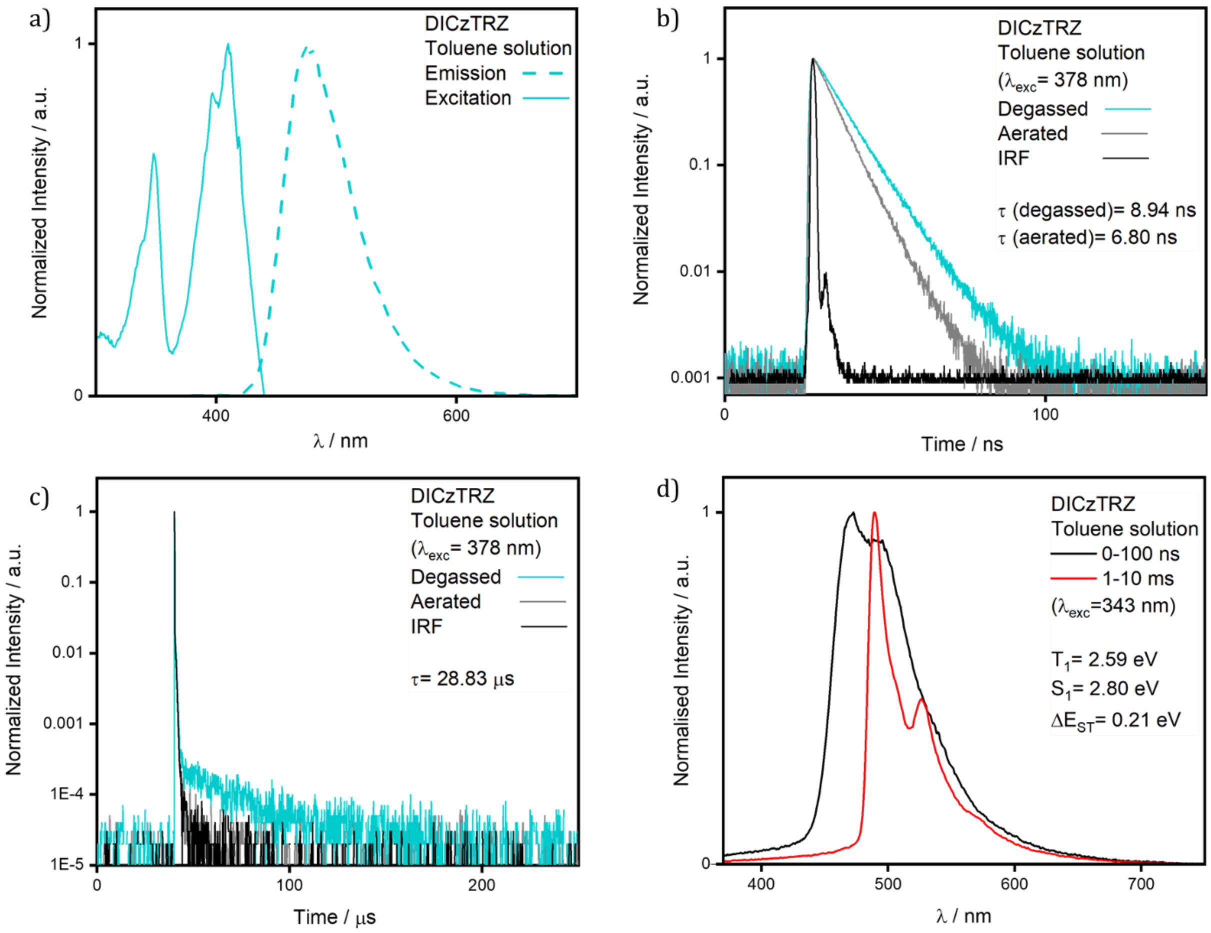

Figure **S4.** a) Emission and excitation spectra of **DICzTRZ** (toluene, 10^{-5} M solutions, λ_{exc} = 360 nm) b) Prompt and c) delayed time-resolved decay of degassed and aerated solutions of **DICzTRZ** (toluene, 10^{-5} M solutions, λ_{exc} = 378 nm). d) Prompt fluorescence spectra at 77 K, and phosphorescence spectra at 77 K in toluene glass (λ_{exc} = 343 nm, prompt and delayed fluorescence spectra were obtained in the 1–100 ns and 1–10 ms time range, respectively).

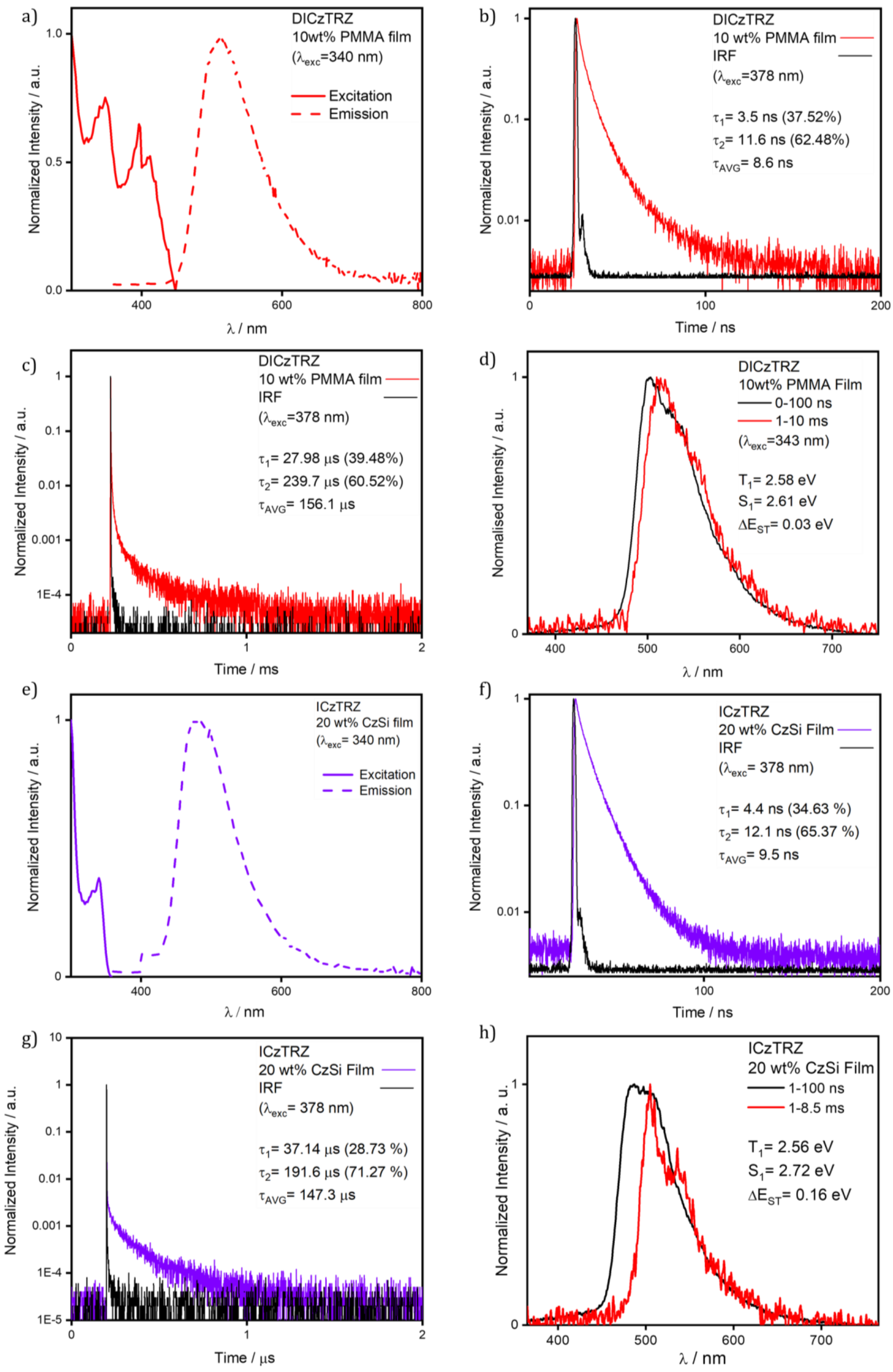
a)
DICzTRZ
10wt% PMMA film
(λexc=340 nm)
Excitation
Emission
Normalized Intensity / a.u.
λ / nm
b)
DICzTRZ
10 wt% PMMA film
IRF
(λexc=378 nm)
τ1= 3.5 ns (37.52%)
τ2= 11.6 ns (62.48%)
τAVG= 8.6 ns
Normalized Intensity / a.u.
Time / ns
c)
DICzTRZ
10 wt% PMMA film
IRF
(λexc=378 nm)
τ1= 27.98 µs (39.48%)
τ2= 239.7 µs (60.52%)
τAVG= 156.1 µs
Normalized Intensity / a.u.
Time / ms
d)
DICzTRZ
10wt% PMMA Film
0-100 ns
1-10 ms
(λexc=343 nm)
T1= 2.58 eV
S1= 2.61 eV
ΔEST= 0.03 eV
Normalised Intensity / a.u.
λ / nm
e)
ICzTRZ
20 wt% CzSi film
(λexc= 340 nm)
Excitation
Emission
Normalized Intensity / a.u.
λ / nm
f)
ICzTRZ
20 wt% CzSi Film
IRF
(λexc= 378 nm)
τ1= 4.4 ns (34.63 %)
τ2= 12.1 ns (65.37 %)
τAVG= 9.5 ns
Normalized Intensity / a.u.
Time / ns
g)
ICzTRZ
20 wt% CzSi Film
IRF
(λexc= 378 nm)
τ1= 37.14 µs (28.73 %)
τ2= 191.6 µs (71.27 %)
τAVG= 147.3 µs
Normalized Intensity / a.u.
Time / µs
h)
ICzTRZ
20 wt% CzSi Film
1-100 ns
1-8.5 ms
T1= 2.56 eV
S1= 2.72 eV
ΔEST= 0.16 eV
Normalized Intensity / a. u.
λ / nm

Figure **S5.** a) Emission and excitation spectra of **DICzTRZ** in spin-coated 10 wt % PMMA film (λ_{exc} = 340 nm). b) Prompt and c) delayed time-resolved decay in spin-coated 20 wt % CzSi film of **DICzTRZ** (λ_{exc} = 378 nm); d) Prompt fluorescence spectra at 77 K, and phosphorescence spectra at 77 K in drop-casted 10 wt % PMMA film (λ_{exc} = 343 nm, prompt and delayed fluorescence spectra were obtained in the 1−100 ns and 1−10 ms time range, respectively); e) emission and excitation spectra of **ICzTRZ** in spin-coated 20 wt % CzSi film (λ_{exc}=340 nm); f) Prompt and g) delayed time-resolved decay in spin-coated 20 wt % CzSi film of **ICzTRZ** (λ_{exc} = 378 nm). h) Prompt fluorescence spectra at 77 K, and phosphorescence spectra at 77 K of **ICzTRZ** in drop-casted 20 wt % CzSi film (λ_{exc} = 343 nm, prompt and delayed fluorescence spectra were obtained in the 1–100 ns and 1–8.5 ms time range, respectively).

Orientation measurements

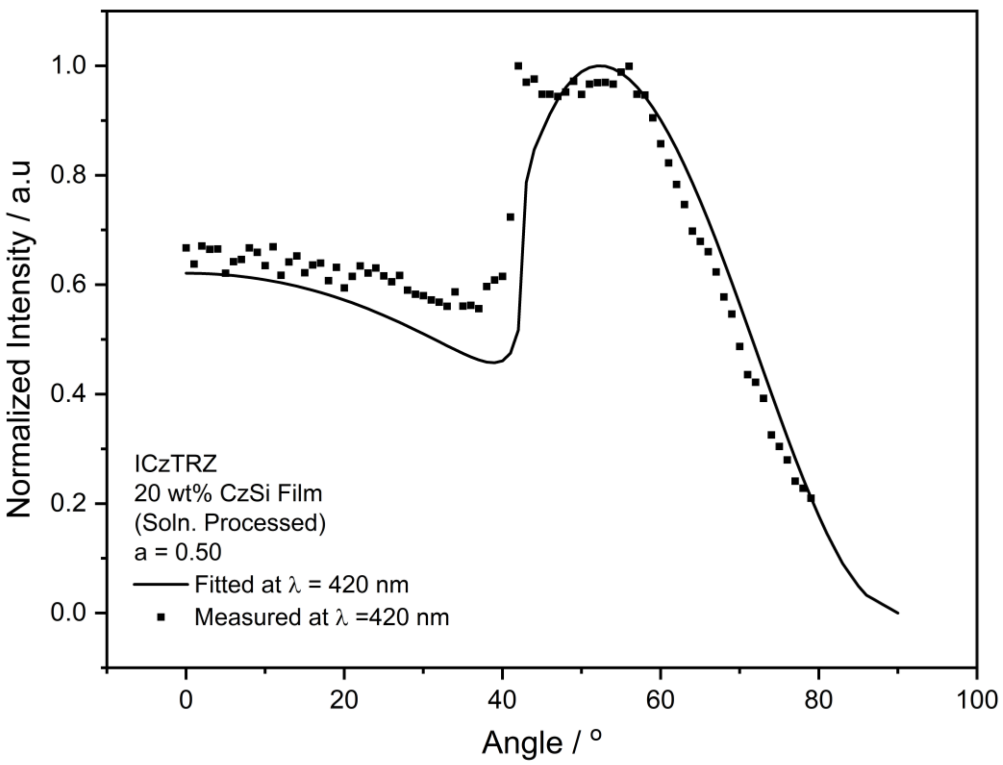

Figure **S6**. Angle-resolved photoluminescence measurement of a solution-processed film of 20 wt % **ICzTRZ** in CzSi. The line shows a fit using the dipole emission model as described above, yielding an anisotropy factor, *a*, of 0.50 (data are taken at λ_{em} = 420 nm).

Device characterization

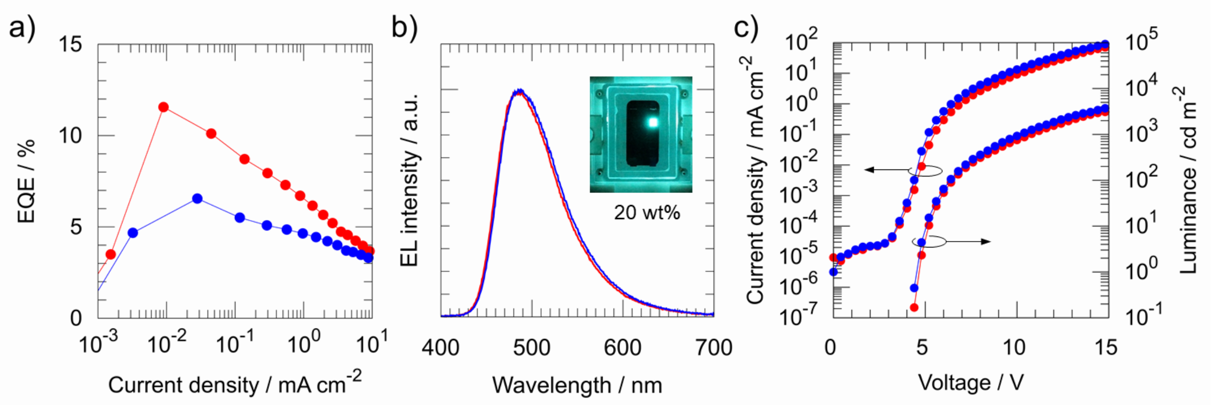

Figure **S7**. Device characteristics of 20 and 30 wt % **ICzTRZ**-based OLEDs, which are represented by red and blue, respectively. a) EQE-current density b) EL spectra and c) current density-voltage-luminance properties.

Device simulation

$$EQE = \gamma\ \eta\ PLQY\ \eta_{out}.$$

γ: charge carrier balance; η_r: radiative exciton fraction; $PLQY$: photoluminescence quantum yield; η_{out}: outcoupling factor.

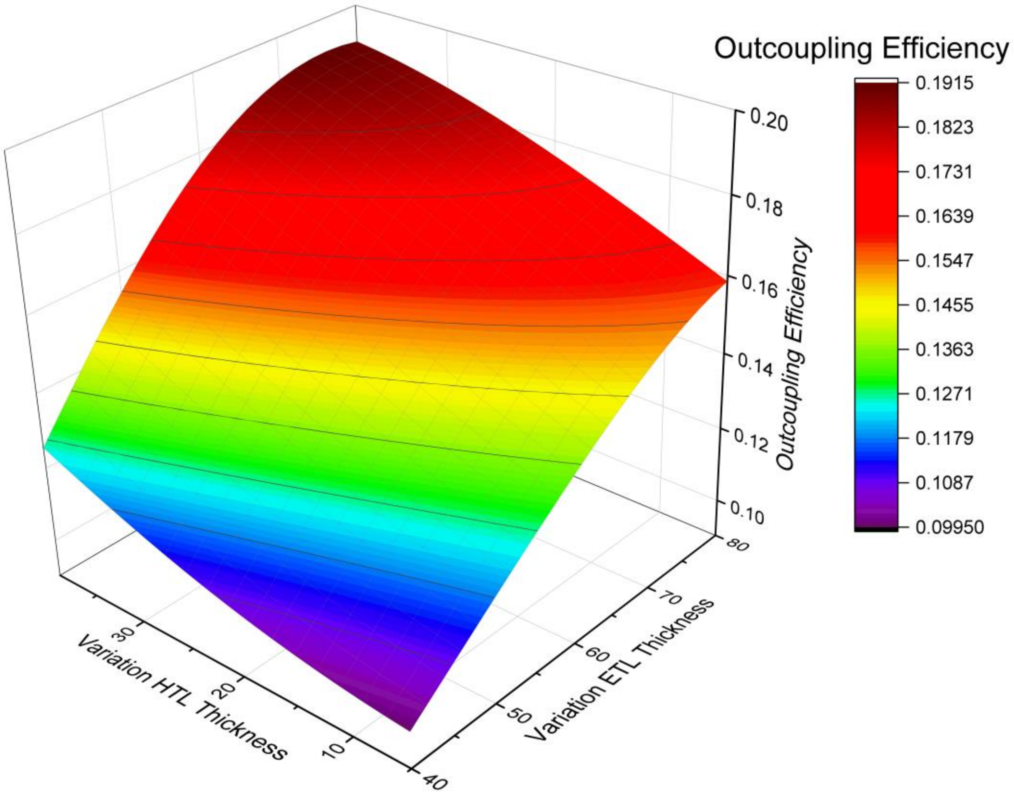

Figure **S8**. Simulated stack optimization with measured emitter orientation of **DICzTRZ**. The maximum outcoupling of 0.1915 is expected with an ETL thickness of 80 nm and a HTL thickness of 40 nm. For **ICzTRZ** as the emitter, slightly different results are expected because of a blue-shifted emission spectrum (and a minute change in orientation).

References

(1) Sharma, N.; Spuling, E.; Mattern, C. M.; Li, W.; Fuhr, O.; Tsuchiya, Y.; Adachi, C.; Bräse, S.; Samuel, I. D. W.; Zysman-Colman, E. *Chem. Sci.* **2019**, *10* (27), 6689–6696. doi:10.1039/c9sc01821b

(2) Connelly, N. G., and Geiger, W. E. *Chem. Rev.* **1996**, *96* (2), 877–910

(3) Crosby, G. A.; Demas, J. N. *J. Phys. Chem.* **1971**, *75* (8), 991–1024. doi:10.1021/j100678a001

(4) Melhuish, W. H. *J. Phys. Chem.* **1961**, *65* (2), 229–235. doi:10.1021/j100820a009

(5) Frisch, M. J.; Trucks, G. W.; Schlegel, H. B.; Scuseria, G. E.; Robb, M. a.; Cheeseman, J. R.; Scalmani, G.; Barone, V.; Petersson, G. a.; Nakatsuji, H.; Li, X.; Caricato, M.; Marenich, a. V.; Bloino, J.; Janesko, B. G.; Gomperts, R.; Mennucci, B.; Hratchian, H. P.; Ortiz, J. V.; Izmaylov, a. F.; Sonnenberg, J. L.; Williams; Ding, F.; Lipparini, F.; Egidi, F.; Goings, J.; Peng, B.; Petrone, A.; Henderson, T.; Ranasinghe, D.; Zakrzewski, V. G.; Gao, J.; Rega, N.; Zheng, G.; Liang, W.; Hada, M.; Ehara, M.; Toyota, K.; Fukuda, R.; Hasegawa, J.; Ishida, M.; Nakajima, T.; Honda, Y.; Kitao, O.; Nakai, H.; Vreven, T.; Throssell, K.; Montgomery Jr., J. a.; Peralta, J. E.; Ogliaro, F.; Bearpark, M. J.; Heyd, J. J.; Brothers, E. N.; Kudin, K. N.; Staroverov, V. N.; Keith, T. a.; Kobayashi, R.; Normand, J.; Raghavachari, K.; Rendell, a. P.; Burant, J. C.; Iyengar, S. S.; Tomasi, J.; Cossi, M.; Millam, J. M.; Klene, M.; Adamo, C.; Cammi, R.; Ochterski, J. W.; Martin, R. L.; Morokuma, K.; Farkas, O.; Foresman, J. B.; Fox, D. J. G16_C01. 2016, p Gaussian 16, Revision C.01, Gaussian, Inc., Wallin

(6) Adamo, C.; Barone, V. *J. Chem. Phys.* **1999**, *110* (13), 6158–6170

(7) Dunning, T. H. *J. Chem. Phys.* **1989**, *90* (2), 1007–1023

(8) Grimme, S. *Chem. Phys. Lett.* **1996**, *259*, 128–137

(9) Hirata, S.; Head-Gordon, M. *Chem. Phys. Lett.* **1999**, *314* (3–4), 291–299. doi:10.1016/S0009-2614(99)01149-5

(10) Moral, M.; Muccioli, L.; Son, W. J.; Olivier, Y.; Sancho-Garcia, J. C. *J. Chem. Theory Comput.* **2015**, *11* (1), 168–177. doi:10.1021/ct500957s

(11) Frischeisen, J.; Yokoyama, D.; Adachi, C. and; Brütting, W. *Appl. Phys. Lett.* **2010**, *96* (7), 1–4. doi:10.1063/1.3309705

(12) Schmidt, T. D.; Lampe, T.; Daniel Sylvinson, M. R.; Djurovich, P. I.; Thompson, M. E.; Brütting, W. *Phys. Rev. Appl.* **2017**, *8* (3), 37001. doi:10.1103/PhysRevApplied.8.037001
